# Endothelial cell–specific LAT1 ablation normalizes tumor vasculature

**DOI:** 10.1172/jci.insight.171371

**Published:** 2024-08-20

**Authors:** Jun-ichi Suehiro, Toru Kimura, Toshiyuki Fukutomi, Hisamichi Naito, Yasuharu Kanki, Youichiro Wada, Yoshiaki Kubota, Nobuyuki Takakura, Hiroyuki Sakurai

**Affiliations:** 1 Department of Pharmacology and Toxicology, Kyorin University School of Medicine, Mitaka, Tokyo, Japan.; 2 Department of Vascular Physiology, Kanazawa University Graduate School of Medical Science, Kanazawa, Ishikawa, Japan.; 3 Laboratory of Clinical Examination and Sports Medicine, Department of Clinical Medicine, Faculty of Medicine, University of Tsukuba, Tsukuba, Ibaraki, Japan.; 4 Isotope Science Center, The University of Tokyo, Bunkyo-ku, Tokyo, Japan.; 5 Department of Anatomy, Keio University School of Medicine, Shinjuku-ku, Tokyo, Japan.; 6 Department of Signal Transduction, Research Institute for Microbial Diseases, Osaka University, Suita, Osaka, Japan.

**Keywords:** Angiogenesis, Vascular biology, Amino acid metabolism, Endothelial cells, Transport

## Abstract

Some endothelial cells in the tumor vasculature express a system L amino acid transporter, LAT1. To elucidate the role of LAT1 in tumor-related endothelial cells, tumor cells were injected into endothelial cell–specific LAT1 conditional knockout mice (*Slc7a5*^flox/flox^; *Cdh5-Cre-ERT2*), and we found that the shape of the tumor vasculature was normalized and the size and numbers of lung metastasis was reduced. TNF-α–induced expression of VCAM1 and E-selectin at the surface of HUVEC, both of which are responsible for enhanced monocyte attachment and premetastatic niche formation, was reduced in the presence of LAT1 inhibitor, nanvuranlat. Deprivation of tryptophan, a LAT1 substrate, mimicked LAT1 inhibition, which led to activation of MEK1/2-ERK1/2 pathway and subsequent cystathionine γ lyase (CTH) induction. Increased production of hydrogen sulfide (H_2_S) by CTH was at least partially responsible for tumor vascular normalization, leading to decreased leakiness and enhanced delivery of chemotherapeutic agents to the tumor.

## Abstract 2

Endothelial specific LAT1 inhibition normalizes tumor vasculature leading to suppression of premetastatic niche formation and improves delivery of anti-tumor chemotherapeutic drugs.

## Graphical abstract

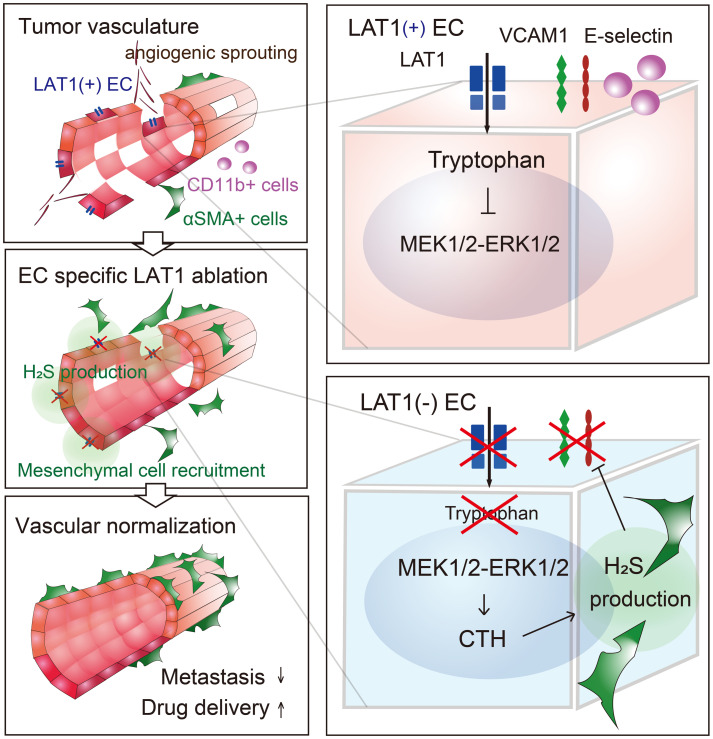

## Introduction

System L amino acid transporter 1 (LAT1; also known as SLC7A5) was independently identified by 2 groups in 1998 (1, 2). LAT1 forms a heterodimer with 4F2hc and transports neutral amino acids including the branched chain amino acids tryptophan and methionine. Because LAT1 has been shown to be selectively expressed in high-grade malignant tumor cells (3, 4), it is regarded as a promising target for cancer therapy. In fact, nanvuranlat (also known as JPH203), a selective LAT1 inhibitor, was reported to suppress proliferation of various cancer cell lines such as prostate, breast, head, and neck cancer cells in both in vitro and in vivo models (5–8). The inhibitor is in a clinical trial for biliary tract cancers in Japan.

In addition to suppressing the growth of cancer cells, there have been reports describing the effect of LAT1 inhibition on tumor angiogenesis, where chemical or genetic inhibition of LAT1 leads to growth suppression of endothelial cells (9, 10). This antiangiogenesis effect together with antiproliferative effects on tumor cells made LAT1 inhibition more attractive for cancer therapy. However, when we administered nanvuranlat to a tumor xenograft model, it did not affect tumor angiogenesis. Starting with this unexpected observation, we decided to investigate the effect of LAT1 inhibition in the endothelial cell of the tumor vasculature in vivo.

Our hypothesis was that inhibiting LAT1 function in endothelial cells in vivo may normalize tumor vasculature and that this effect rather than inhibiting angiogenesis is the main effect of LAT1 inhibition in endothelial cells. If so, inhibiting LAT1 in cancer patients may suppress tumor by (a) directly suppressing tumor growth, (b) disrupting tumor related endothelial cell activation resulting in decreased premetastatic niche formation, leading to less distant metastasis, and (c) improving delivery of chemotherapeutic agents to cancer cells by normalizing vascular function.

## Results

### Tumor vessels expressed LAT1 and endothelial cell–specific LAT1 ablation altered tumor vessel formation in shape and inhibited spontaneous lung metastasis.

**Figure 1:**
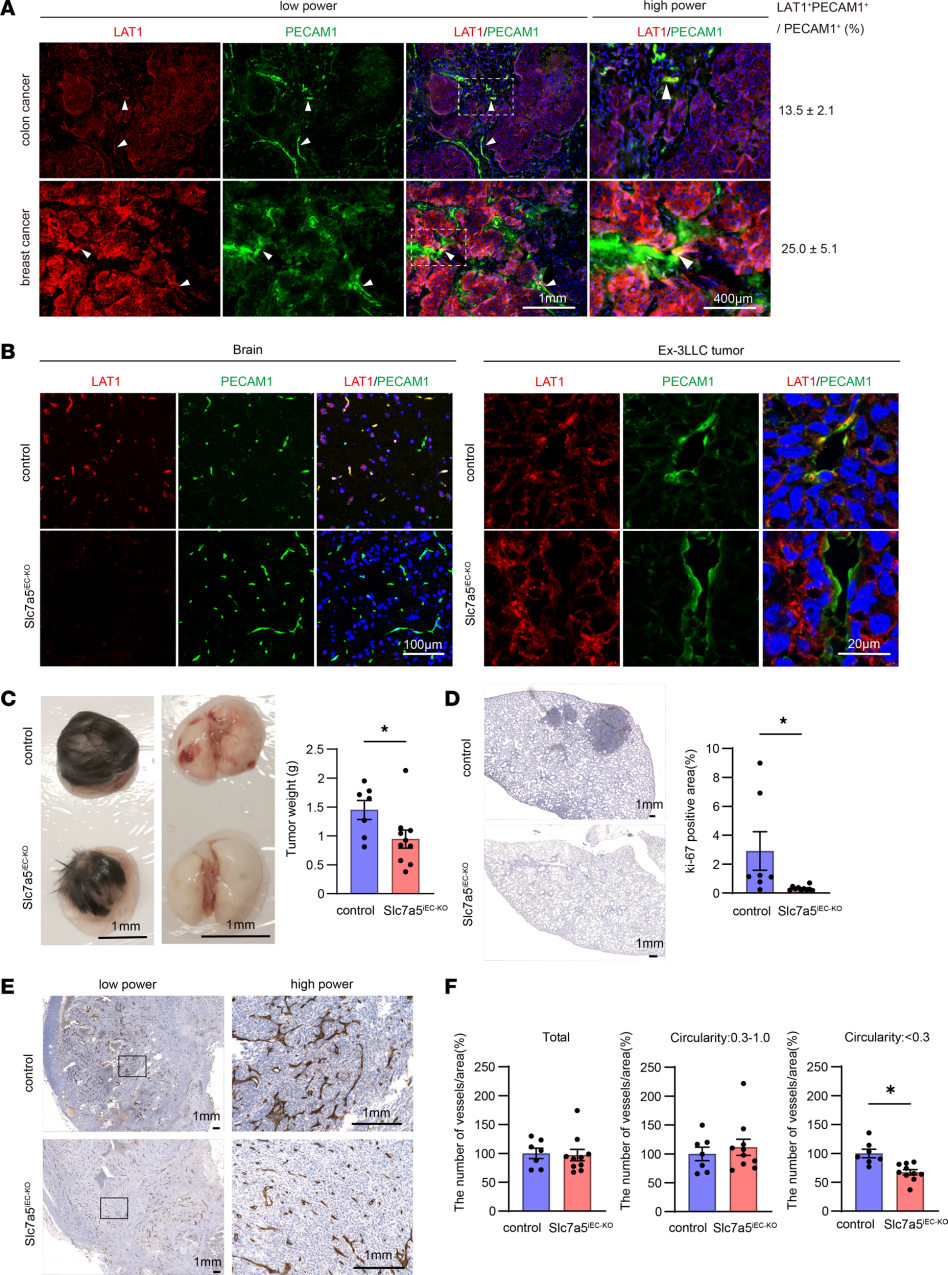
Endothelial cell–specific LAT1 ablation decreased transplanted tumor growth, spontaneous lung metastasis, and tumor vessel deformation. (**A**) Paraffin-embedded human colon or breast cancer tissue was stained with LAT1 and PECAM1 antibodies. White arrow heads indicate LAT1^+^PECAM1^+^ cells. The high-power images correspond to dotted rectangle areas in the low power. The numbers indicate percentages (mean ± SEM, *n* = 24) of LAT1^+^PECAM1^+^ cells to total PECAM1^+^ endothelial cells. Scale bars: 1 mm (low-power images) and 400 μm (high-power images). (**B**) Brain or Ex-3LLC solid tumor slices from control or Slc7a5^iEC–KO^ mice after 4 weeks of Ex-3LLC injection were stained with LAT1 and PECAM1 antibodies. The representative images were counterstained with DAPI. Scale bars: 100 μm (brain) and 20 μm (Ex-3LLC tumor).(**C**) Macroscopic appearance of Ex-3LLC solid tumor and spontaneous lung metastasis after 4 weeks of Ex-3LLC injection into mice. Tumor weight (mean ± SEM) in control or Slc7a5^iEC–KO^ mice was quantified from indicated independent experiments (control; *n* = 7, Slc7a5^iEC–KO^ mice; *n* = 10). Scale bars: 1 cm. (**D**) The representative ki-67 staining of the lung was shown in left panel. Bar graph showed the ki-67 staining area (mean ± SEM) in the lung of mice (control; *n* = 7, Slc7a5^iEC–KO^ mice; *n* = 10). Scale bars: 1 mm. (**E**) PECAM1-stained tumor vessels in the primary solid tumor area after 4 weeks of Ex-3LLC cell injection. The high-power images corresponded to rectangle areas in the low power. Scale bars: 1 mm (low-power images) and 10 mm (high-power images). (**F**) Shape of tumor vasculatures from PECAM1 staining were classified by circularity into low or high circularity group. Bar graphs indicated the number of vessels (mean ± SEM) per area (control; *n* = 7, Slc7a5^iEC–KO^ mice; *n* = 10). *P* values were determined by 2-tailed, unpaired *t* test. *P < 0.05.

We first examined LAT1 expression in tumor blood vessels in human colon and breast cancer tissue sections. In the colon cancer slide, LAT1 was barely detectable in tumor cells but was sparsely found in tumor vessels (Figure 1A, arrow heads, upper panel) while LAT1 was expressed in both tumor cells and vessels in the breast cancer slide (Figure 1A, arrow heads, bottom panel). PECAM1^+^LAT1^+^ cells accounted for 13.5% or 25.0% of total PECAM1^+^ cells in the colon and breast cancer tissue slides, respectively. Although we have not extensively examined other human cancer samples, these results indicate that expression of LAT1 in the tumor vasculature appeared clinically relevant.

To examine whether LAT1 contributes to vascular function in vivo, we have generated *Slc7a5*^flox/flox^; *Cdh5-Cre-ERT2* mice (Details of gene targeting were shown in Supplemental Figure 1; supplemental material available online with this article; https://doi.org/10.1172/jci.insight.171371DS1). Because endothelial cell–specific expression of Cdh5 has been demonstrated (11, 12), we decided to use a *Cdh5* promoter rather than a *Tie2* promoter, which drives Cre expression not only in endothelial cells but also in myeloid-derived cells (13, 14). According to Tărlungeanu et al., in WT animals, Slc7a5 is expressed considerably in the plasma membrane of the endothelial cells of the blood brain barrier during development and in adulthood but it is mostly undetectable in other normal tissues (15), so we compared the expression of Slc7a5 in the blood brain barrier in control mice with that of conditional knockout mice. After 2 weeks of tamoxifen administration to the mice, the endothelial cell–specific removal of Slc7a5 was confirmed in the brain (Figure 1B, left panel). Hereafter we refer to the mice as endothelial cell–specific Slc7a5 conditional knockout mice or Slc7a5^iEC–KO^ mice. The Slc7a5^iEC–KO^ mice exhibited normal weight and size but their limbs trembled, as reported with other endothelial cell–targeted *Slc7a5* conditional knockout mice crossed with LAT1-floxed mice with *Tie2*-Cre mice (15).

To elucidate roles of LAT1 in tumor progression and metastasis, Ex-3 Lewis lung carcinoma (Ex-3LLC) cells were subcutaneously injected into Slc7a5^iEC–KO^ mice. The Ex-3LLC cells were known to form spontaneous lung metastasis after subcutaneous transplantation (16, 17). LAT1 positive endothelial cells were detected in the tumor vasculature in control mice but not in Slc7a5^iEC–KO^ mice (Figure 1B, right panel). After 4 weeks, the size of the primary tumor and the spontaneous lung metastasis were prominently suppressed in Slc7a5^iEC–KO^ mice compared with what was observed in control mice (Figure 1, C and D). At the primary tumor site, tumor vessels in Slc7a5^iEC–KO^ mice showed distinct morphology compared with control mice (Figure 1E). On the other hand, endothelial cell–specific knockout of LAT1 did not affect the shape of blood vessels in brain, lung, heart, kidney, and liver in the absence of tumor (Supplemental Figure 2). Notably, there is no difference in the number of tumor vessels between Slc7a5^iEC–KO^ and control mice (Figure 1F, left panel). However, as opposed to those in control mice, which showed heterogenous and abnormally branched vessels with low circularity of typical tumor vasculature, tumor vessels in Slc7a5^iEC–KO^ mice had uniform density without truncated branches, resulting in high circularity, which resembled the normal vessels (Figure 1F, middle and right panels).

### Intracellular tryptophan starvation caused by LAT1 inhibition suppressed inflammatory adhesion molecule expression via MEK1/2-ERK1/2 activation.

**Figure 2:**
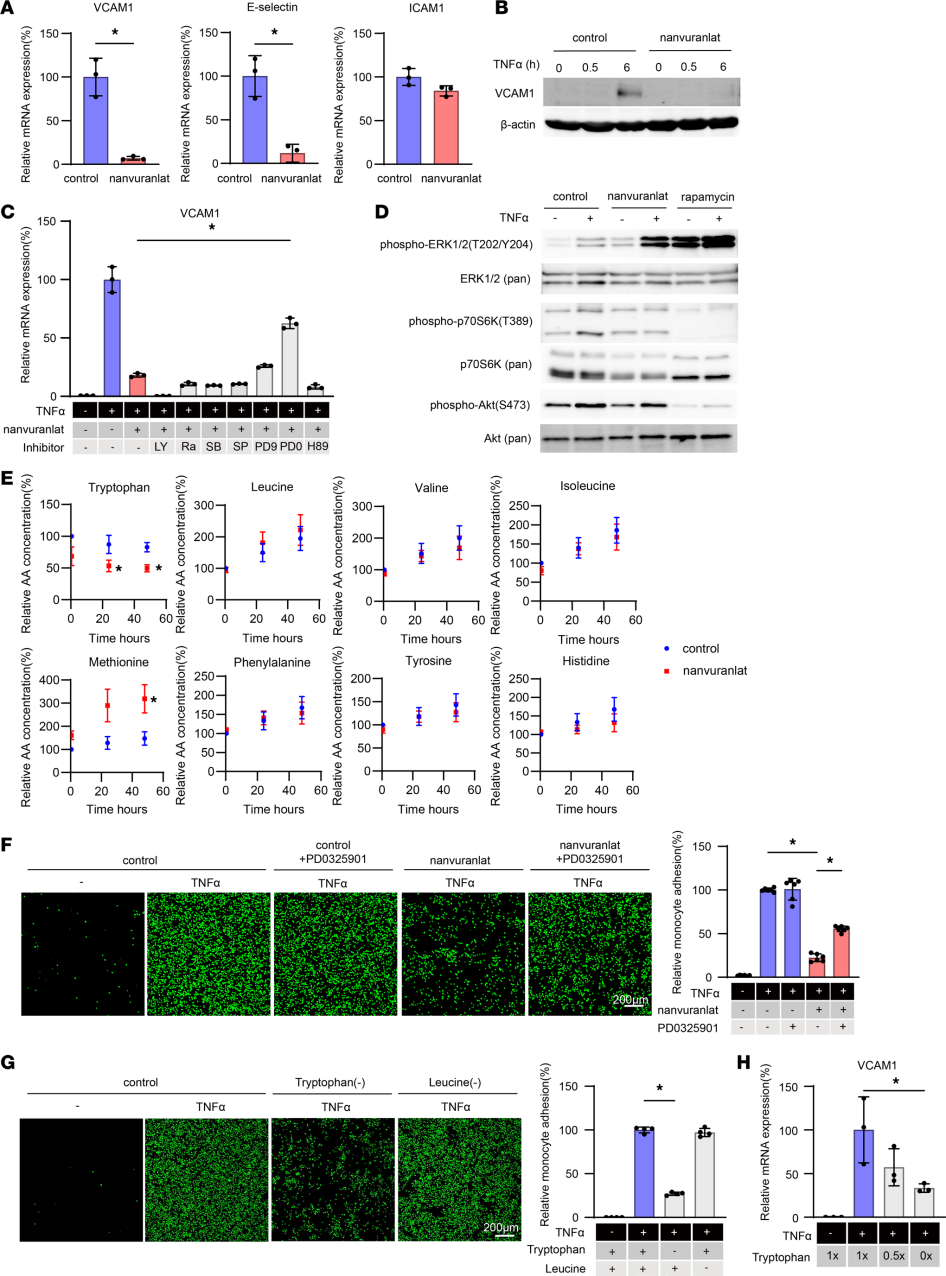
LAT1 inhibition led to a decrease in intracellular tryptophan levels, resulting in suppression of TNF-α–mediated VCAM1 expression and monocyte adhesion via MEK1/2-ERK1/2 signaling cascade. (**A**) qPCR analysis of VCAM1, E-selectin, and ICAM1 mRNA expression in 10 ng/mL TNF-α–stimulated HUVEC with 100 μM captisol or nanvuranlat. (**B**) Western blot analysis of VCAM1 expression in TNF-α–stimulated HUVEC with 100 μM Captisol or nanvuranlat. β-actin was used as a loading control. Blots provided together were set up in parallel at the same time. (**C**) qPCR analysis of TNF-α–mediated VCAM1 mRNA expression treated with indicated signal transduction inhibitors. The inhibitor name and concentration were LY, 50 μM LY294002: Ra, 1 μM rapamycin; SB, 20 μM SB203580; SP, 50 μM SP600125; PD9, 50 μM PD98059; PD0, 50 μM PD0325901; and H89,20 μM H-89. (**D**) Western blot analysis of phosphorylation of indicated proteins in TNF-α–stimulated HUVEC for 10 minutes with 100 μM Captisol, 100 μM nanvuranlat, or 1 μg/mL rapamycin. Blots provided together were set up in parallel at the same time. (**E**) Measurement of intracellular amino acid concentration by LC-MS/MS analysis in 100 μM Captisol (vehicle control) or nanvuranlat treated HUVEC. Graph showed mean ± SEM (*n* = 4) of intracellular amino acid concentration in vehicle control (blue circle) or nanvuranlat (red square) condition. (**F**) U937 monocyte adhesion to HUVEC in the presence of Captisol, nanvuranlat, and/or PD0325901. Graph showed mean ± SD (*n* = 6). (**G**) U937 monocyte adhesion to HUVEC in the tryptophan or leucine deprivation media. Graph showed mean ± SD (*n* = 4). (**H**) qPCR analysis of VCAM1 mRNA expression in HUVEC cultured in tryptophan-starved MCDB131 media. (**A**) *P* values were determined by 2-tailed, unpaired *t* test. (**E**) *P* values were determined by 1-way ANOVA with Tukey’s multiple comparisons test compared to control at 0.5 hour. (**C** and **F**–**H**) *P* values were determined by 1-way ANOVA with Tukey’s multiple comparisons test. *P < 0.05. Scale bars: 200 μm.

To dissect the molecular mechanism underlying the inhibition of lung metastatic colonization in Slc7a5^iEC–KO^ mice, effect of LAT1 inhibition on endothelial cell gene expression and function was investigated in vitro using human umbilical vein endothelial cells (HUVEC) treated with proinflammatory cytokines TNF-α or IL1-β, both of those have been shown to exist in the tumor microenvironment (18, 19) in the presence or absence of nanvuranlat, a selective LAT1 inhibitor. Because expression of inflammatory adhesion molecules in the lung vascular bed is essential for lung metastasis, the change in expression of such molecules was evaluated by real-time qPCR. Both VCAM1 and E-selectin, but not ICAM1, were strongly inhibited by nanvuranlat in TNF-α or IL1-β–treated HUVEC (Figure 2A and Supplemental Figure 3). Along the same line, VCAM1 induction by TNF-α in protein level was absolutely blocked by nanvuranlat (Figure 2B). Downstream of TNF-α, nuclear translocation, but not phosphorylation, of NF-κB, a determinant of VCAM1 induction, was suppressed by LAT1 inhibition (Supplemental Figure 4). Among 7 types of signal transduction inhibitors tested in TNF-α–treated HUVEC in the presence of nanvuranlat, PD98059 and PD0325901, selective MEK1/2-ERK1/2 inhibitors, restored VCAM1 expression (Figure 2C). TNF-α–mediated phosphorylation of ERK1/2 (T202/Y204) apparently increased by nanvuranlat compared with the control, while TNF-α–induced phosphorylation of p70S6K(T389) was cancelled by nanvuranlat and phosphorylation of Akt (S473) was not affected by LAT1 inhibition (Figure 2D). This is in contrast with rapamycin, which suppressed phosphorylation of p70S6K(T389) and Akt (S473) while promoting phosphorylation of ERK1/2 (T202/Y204) (Figure 2D). Thus, it is likely that increased phosphorylation of ERK1/2 by LAT1 inhibition took different signaling pathway from rapamycin treatment. Taken together, these results suggest that MEK1/2-ERK1/2 activation was essential for nanvuranlat-mediated suppression of VCAM1 transcriptional induction.

Because LAT1 is an amino acid transporter, the change in intracellular amino acid concentration by nanvuranlat treatment was globally analyzed by liquid chromatography with tandem mass spectrometry (LC-MS/MS). The concentration of tryptophan, a substrate of LAT1, continuously decreased after 30 minutes of nanvuranlat treatment, while that of methionine, one of the sulfur-containing amino acids, increased after 24 hours of the treatment. (Figure 2E). Among other sulfur-containing amino acids and metabolites, cystathionine was decreased by nanvuranlat (Supplemental Figure 5).

Consistent with the increased expression levels of adhesion molecules on the endothelial cell, TNF-α stimulated monocyte adhesion to HUVEC in the absence of nanvuranlat while the number of surface-attached monocytes was significantly reduced in the presence of nanvuranlat and PD0325901 rescued its suppression (Figure 2F). Tryptophan deprivation mimicked the effect of nanvuranlat, which decreased TNF-α-mediated monocyte adhesion to HUVEC, while leucine deprivation did not affect the process (Figure 2G). In real-time qPCR analysis, tryptophan deprivation attenuated VCAM1 induction in inverse dose-dependent manner (Figure 2H).

### LAT1 inhibition led to cystathionine-γ-lyase–mediated hydrogen sulfide production, which inhibited U937 monocyte adhesion to HUVEC.

**Figure 3:**
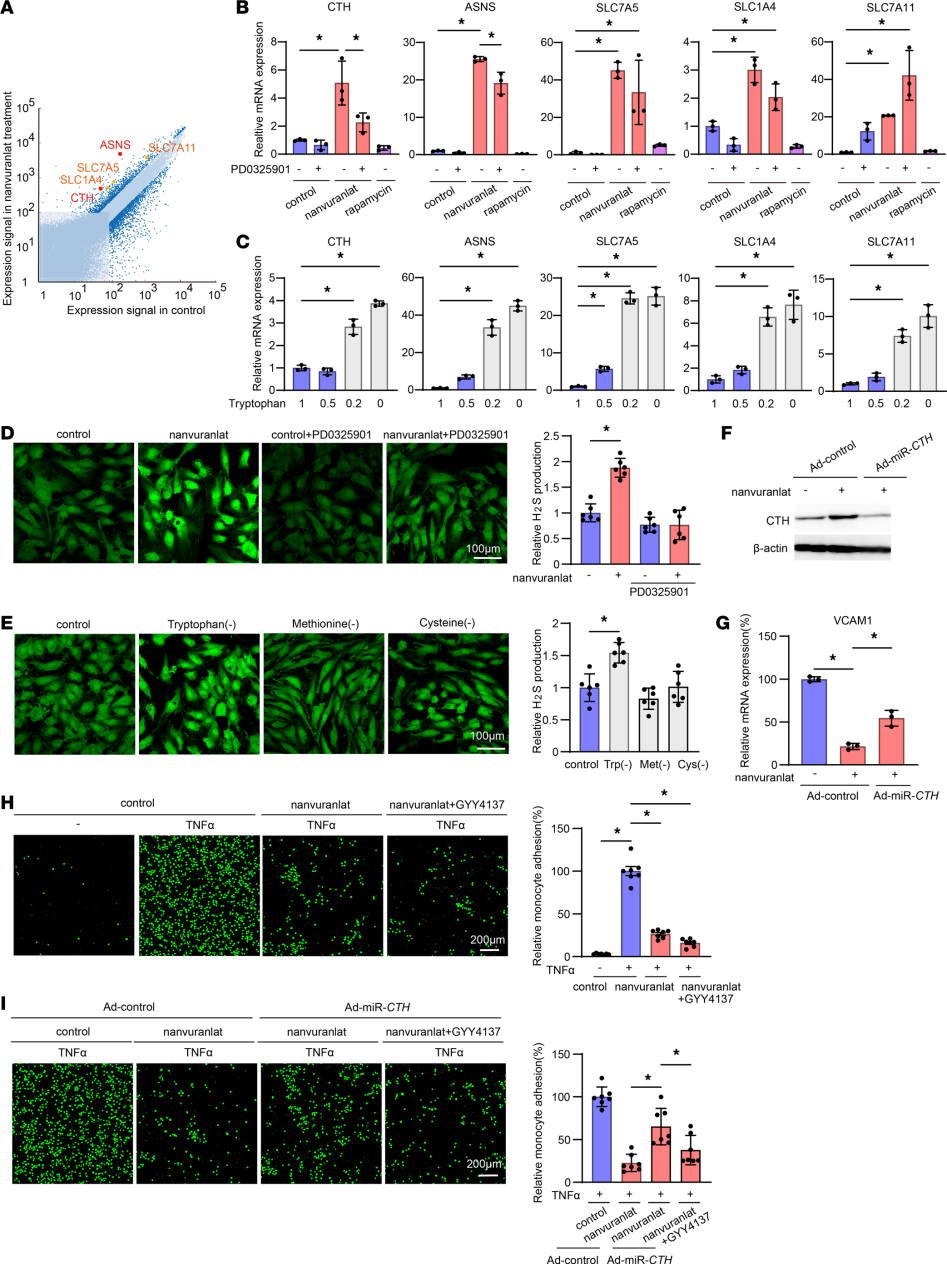
LAT1 inhibition led to CTH-mediated H_2_S production, which impaired U937 monocyte adhesion to HUVEC. (**A**) DNA microarray analysis in HUVEC treated with 100 μM captisol or nanvuranlat for 48 hours. Dark blue dots indicate genes with a greater-than 2-fold up- or downregulation due to nanvuranlat. Red or orange dots showed amino acid metabolic genes or amino acid transporters from GO analysis. (**B**) qPCR expression analysis of HUVEC cultured in 100 μM captisol (control) or nanvuranlat in the presence or absence of 50 μM PD0325901 or 1 μg/mL rapamycin. (**C**) qPCR expression analysis of HUVEC cultured in 1, 0.5, 0.2, or 0 × tryptophan restriction media. (**D**) Quantification of H_2_S production in HUVEC cultured in EGM2 supplemented with 100 μM captisol or nanvuranlat in the presence or absence of 50 μM PD0325901. HUVEC were loaded with HSip1-DA to visualize H_2_S production. Bar graph showed mean ± SD (*n* = 6). (**E**) H_2_S production in amino acid starved MCDB131 media. Graph showed mean ± SD (*n* = 6). (**F**) Western blot analysis of CTH expression in Ad-control or Ad-miR-*CTH*–infected HUVEC in the presence or absence of 100 μM captisol or nanvuranlat. β-actin was used as a loading control. Blots provided together were set up in parallel at the same time. (**G**) qPCR analysis of VCAM1 expression in Ad-control or Ad-miR-*CTH*–infected HUVEC under 10 ng/mL TNF-α treatment for 4 hours. (**H**) U937 monocyte adhesion to GYY4137-loaded HUVEC in 100 μM Captisol or nanvuranlat. Bar graph showed mean ± SD (*n* = 7). (**I**) Monocyte adhesion to Ad-miR-*CTH*–infected HUVEC in 100 μM captisol or nanvuranlat. Bar graph showed mean ± SD (*n* = 7). *P* values were determined by 1-way ANOVA with Tukey’s multiple comparisons test. *P < 0.05. Scale bars: 100 μm (**D** and **E**) and 200 μm (**H** and **I**).

To elucidate the molecular mechanism for nanvuranlat-mediated VCAM1 suppression, change in HUVEC transcriptome in the presence or absence of nanvuranlat was evaluated by DNA microarray. The data were filtered by 2-fold upregulation or downregulation by the nanvuranlat treatment. GO analysis showed that amino-acid transporter and metabolizing enzyme cluster was significantly changed by nanvuranlat. Cystathionine-γ-lyase (CTH), asparagine synthetase (ASNS), SLC7A5, the alanine, serine, and cysteine transporters SLC1A4 (also known as ASCT1) and the glutamate/cystine antiporter (SLC7A11; also known as xCT) were the differentially expressed genes in the cluster (Figure 3A). Real-time qPCR analysis validated microarray data. Of these 5 genes, which were not induced by rapamycin, only CTH and ASNS upregulation was suppressed by PD0325901 (Figure 3B). Tryptophan deprivation also augmented expression of the above-mentioned 5 genes in an inverse dose-dependent manner (Figure 3C). Thus, these results suggest that inhibition of LAT1 by nanvuranlat depleted intracellular tryptophan, which, in turn, induced the expression of 5 amino acid metabolism–related genes, and that only CTH and ASNS were downstream of ERK1/2. Because CTH was essential for the sulfur-containing amino acid metabolic process and its catalyzed product, hydrogen sulfide (H_2_S), is an angiogenic and antiinflammatory gas transmitter (20, 21), we focused on CTH function in endothelial cells.

Consistent with the gene expression data, HUVEC increased H_2_S production in the presence of nanvuranlat and this increase was cancelled by PD0325901(Figure 3D). Tryptophan deprivation significantly upregulated H_2_S production, while methionine or cysteine deprivation did not (Figure 3E). Addition of tryptophan to culture media reversed nanvuranlat-mediated H_2_S production, while that of leucine, methionine, or cysteine did not (Supplemental Figure 6). In the same context, only addition of tryptophan attenuated the antiinflammatory effect observed with nanvuranlat (Supplemental Figure 7). Along the same line, leucine, methionine, or cysteine deprivation only slightly suppress TNF-α–mediated monocyte adhesion to HUVEC, compared with tryptophan deprivation (Supplemental Figure 8). When CTH upregulation was inhibited by introducing a miRNA construct against *CTH* (Ad-miR-*CTH*) to TNF-α–treated HUVEC, nanvuranlat mediated VCAM1 suppression was reversed by about 50% (Figure 3, F and G).

In a monocyte adhesion assay, addition of GYY4137, an H_2_S donor, to nanvuranlat-treated HUVEC did not show much effect (Figure 3H), while the same treatment significantly reversed the effect of CTH knockdown in the presence of nanvuranlat (Figure 3I), suggesting that the antiinflammatory effect of LAT1 inhibition is largely explained by increased production of H_2_S. However, it is of note that addition of GYY4137 or Ad-miR-*CTH* in the absence of LAT1 inhibition showed only a slight effect on monocyte adhesion or VCAM1 expression (Supplemental Figures 9–12), indicating that H_2_S production plays a significant role in regulating the inflammatory response of endothelial cells only in the context of LAT1 inhibition. Taken together, these data suggested that H_2_S production downstream of CTH is at least partially responsible for the decreased monocyte attachment and VCAM1 expression observed in nanvuranlat-treated HUVEC.

### Endothelial cell–specific LAT1 ablation suppressed CD11b-positive myeloid cell recruitment and endothelial VCAM1 expression in the premetastatic lung, leading to attenuated lung metastatic colonization.

**Figure 4:**
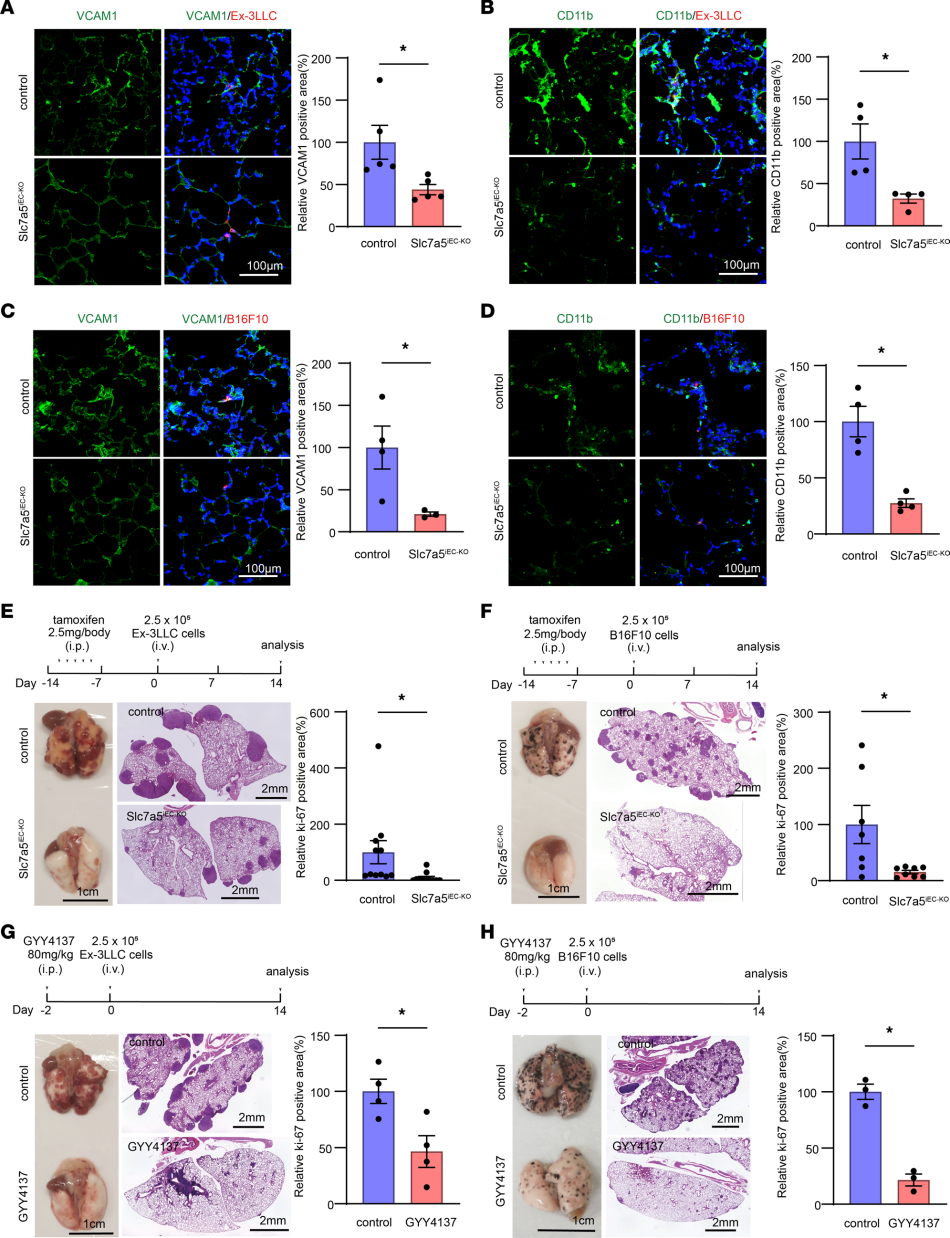
Endothelial cell–specific LAT1 ablation suppressed CD11b-positive myeloid cell recruitment and endothelial VCAM1 expression in the premetastatic lung, inhibiting Ex-3LLC or B16F10 lung metastasis. (**A**) Immunostaining of VCAM1 in lungs from control or Slc7a5^iEC–KO^ mice intravenously injected with fluorescent-labeled Ex-3LLC cells. Nuclei were counterstained with DAPI (blue). Bar graph represented the numbers of VCAM1-positive cells (fluorescence intensity more than background)/hpf counted in 5 randomly selected slices and normalized to that of control. (**B**) Immunostaining of CD11b in lungs from control or Slc7a5^iEC–KO^ mice intravenously injected with fluorescent-labeled Ex-3LLC cells. The numbers of CD11b cells/hpf were counted in 4 randomly selected slices and normalized to that of control. (**C**) Immunostaining of VCAM1 in lungs from control or Slc7a5^iEC–KO^ mice intravenously injected with fluorescent-labeled B16F10 cells (control; *n* = 4, Slc7a5^iEC–KO^ mice; *n* = 3). (**D**) Immunostaining of CD11b in lungs removed from control or Slc7a5^iEC–KO^ mice intravenously injected with fluorescent-labeled B16F10 cells (control; *n* = 4, Slc7a5^iEC–KO^ mice; *n* = 4). (**E**) Lung metastasis model by intravenous injection of Ex-3LLC cells in control or Slc7a5^iEC–KO^ mice. Time course of tamoxifen administration and cancer cell injection are indicated at top. Middle images indicate representative images of whole lung and histological sections of the lung with hematoxylin and eosin staining. Scale bars (**E**–**H**): 1 cm (left); 2 mm (right). The ratio of ki-67 positive area in the bar graph was normalized to the control mice (= 100%) (mean ± SEM, control; *n* = 11, Slc7a5^iEC–KO^ mice; *n* = 12). (**F**) Lung metastasis model by intravenous injection of B16F10 cells in control or Slc7a5^iEC–KO^ mice (control; *n* = 7, Slc7a5^iEC–KO^ mice; *n* = 8). (**G**) Lung metastasis model by intravenous injection of Ex-3LLC cells in control or GYY4137 loaded mice (control; *n* = 4, GYY4137 treated mice; *n* = 4). The ratio of ki-67 positive area was normalized to the control mice (= 100%). (**H**) Lung metastasis model by intravenous injection of B16F10 cells in GYY4137 loaded mice (control; *n* = 3, GYY4137 treated mice; *n* = 3). Bar graph showed mean ± SEM. *P* values were determined by 2-tailed, unpaired *t* test. *P < 0.05. Scale bars (**A**–**D**): 100 μm.

A previous report suggests that inflammatory adhesion molecule expression and CD11b-positive myeloid cell recruitment were essential for premetastatic niche formation before the arrival of tumor cells (22). VCAM1 expression of the lung endothelial cells and CD11b-positive myeloid cell accumulation at the surface of lung endothelial cells were decreased in Ex-3LLC or B16F10 intravenously injected Slc7a5^iEC–KO^ mice compared with the same tumor-injected control mice (Figure 4, A–D). The number and size of the metastatic colony was also significantly smaller in Slc7a5^iEC–KO^ mice than in control mice (Figure 4, E and F). Administration of GYY4137 to WT mice before the intravenous injection of Ex-3LLC and B16F10 tumor cells attenuated lung metastatic colonization, which resembled the tumor metastasis inhibition observed in Slc7a5^iEC–KO^ mice (Figure 4, G and H). Collectively, these results suggest that lung metastasis of Ex-3LLC or B16F10 cells were inhibited by increased H_2_S production in Slc7a5^iEC–KO^ mice.

### Increased H_2_S production in endothelial cells of Slc7a5^iEC–KO^ mice promoted tumor vessel normalization and mesenchymal cell recruitment.

It has been reported that tumor-related blood vessels often lack perivascular mesenchymal cells, which may be related to abnormal shape of tumor vasculature and is translated into increased leakiness of the tumor vasculature becoming the obstacle to delivery of chemotherapeutic agents to the tumor (23).

**Figure 5:**
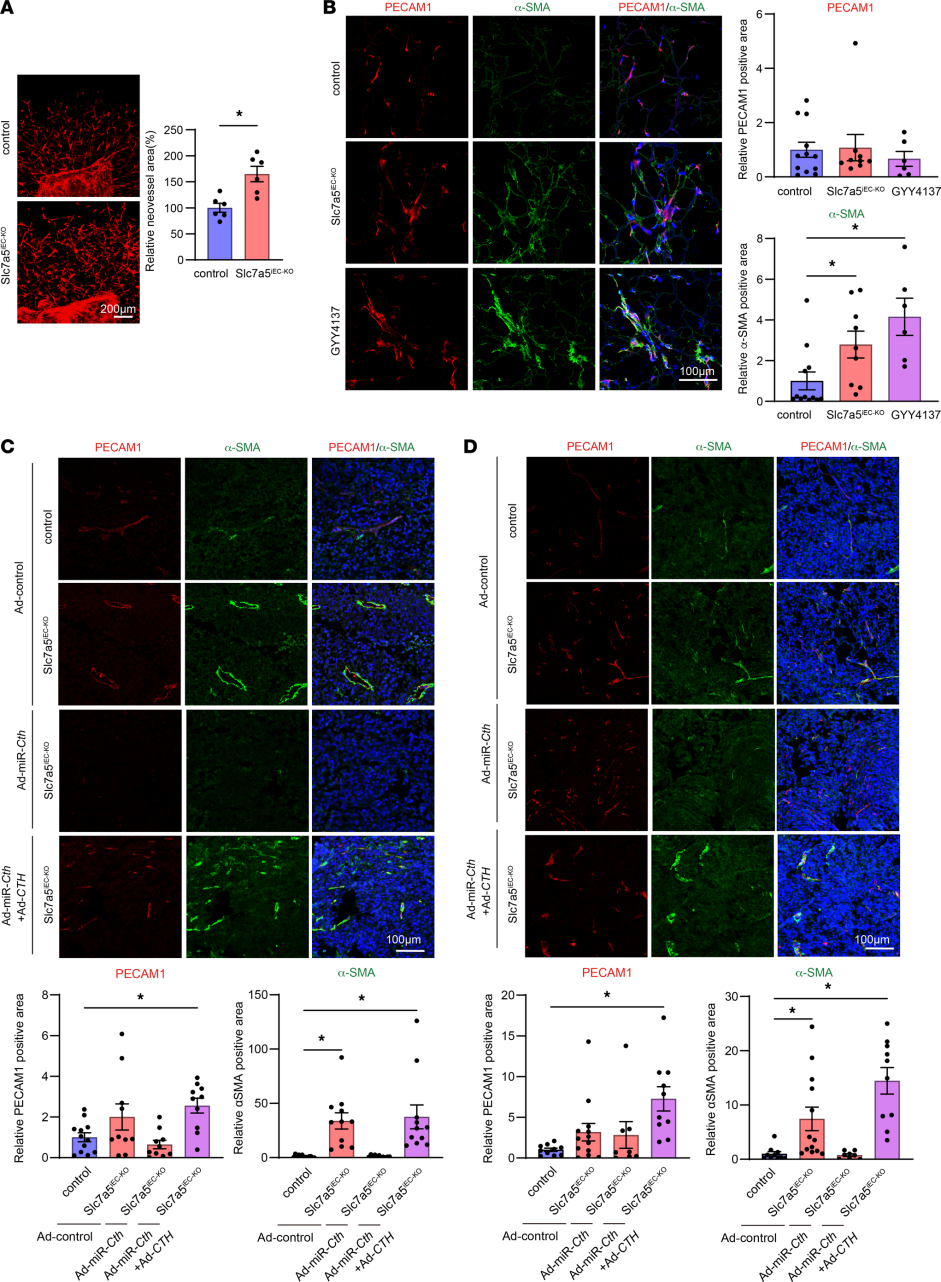
Slc7a5^iEC–KO^ mice promoted tumor vessel normalization and mesenchymal cell migration via Cth induction. (**A**) Aortic ring assay in control or Slc7a5^iEC–KO^ mice. Area of phalloidin-positive cells (mean ± SEM) was calculated from 6 randomly selected images derived from each mouse and normalized to that of control mice (the mean of the control mice was calculated as 100%). (**B**) Matrigel plug assay in control, Slc7a5^iEC–KO^ or GYY4137 treated C57BL/6J mice (mean ± SEM, control; *n* = 12, Slc7a5^iEC–KO^ mice; *n* = 9, GYY4137-treated mice; *n* = 6). Nuclei were counterstained with DAPI (blue). (**C**) Immunofluorescent analysis of PECAM1 and α-SMA–positive cell localization in Ex-3LLC tumor in Ad-control, Ad-miR-*Cth*, or Ad-miR-*Cth* + Ad-*CTH* infected mice at day 16. Data indicated mean ± SEM (control + Ad-control; *n* = 12, Slc7a5^iEC–KO^ +Ad-control; *n* = 10, Slc7a5^iEC–KO^ +Ad-miR-*Cth*; *n* = 10, Slc7a5^iEC–KO^ + Ad-miR-*Cth* and Ad-*CTH*; *n* = 10). (**D**) Immunofluorescent analysis of PECAM1 and α-SMA-positive cell localization in B16F10 tumor at day 15 in Ad-control, Ad-miR-*Cth*, Ad-miR-*Cth* + Ad-*CTH* infected mice. Data indicated mean ± SEM (control + Ad-control; *n* = 10, Slc7a5^iEC–KO^ +Ad-control; *n* = 13, Slc7a5^iEC–KO^ +Ad-miR-*Cth*; *n* = 8, Slc7a5^iEC–KO^ +Ad-miR-*Cth* and Ad-*CTH*; *n* = 10). (**A**) *P* values were determined by 2-tailed, unpaired *t* test. (**B**–**D**) *P* values were determined by 1-way ANOVA with Holm-Šídák’s multiple comparisons test compared to control. *P < 0.05. Scale bars: 200 μm (**A**); 100 μm (**B**–**D**).

We suspected that morphological normalization of tumor vessel in Slc7a5^iEC–KO^ mice (Figure 1) may be accompanied by recruitment of more perivascular mesenchymal cells. Thus, to evaluate the degree of mesenchymal cell attraction near newly formed blood vessels, an aortic ring assay was performed. The number of phalloidin-positive migrating cells, appearing to be perivascular mesenchymal cells, was increased in Slc7a5^iEC–KO^ mice (Figure 5A), suggesting that blood vessels sprouting from the aortic ring of Slc7a5^iEC–KO^ were structurally more normal than those from WT mice. Similar results were obtained with Matrigel plug assay; the number of α-smooth muscle actin–positive (α-SMA–positive) cells was increased in Slc7a5^iEC–KO^ mice compared with control mice, while the number of migrating endothelial cells was not different between them. GYY4137 augmented migration of α-SMA–positive mesenchymal cells in WT mice, suggesting that increased production of H_2_S plays an important role in attracting mesenchymal cells to blood vessels (Figure 5B). In fact, GYY4137 augmented fibroblast migration for 24 hours after a scratch in dermal fibroblast cell culture (Supplemental Figure 13). To further evaluate the relationship between endothelial cells and fibroblasts, coculture migration assay in cell culture inserts was performed. Nanvuranlat augmented migration of fibroblast cells, while CTH knockdown in endothelial cells attenuated fibroblast migration (Supplemental Figure 14). Similarly, nanvuranlat treatment increased the number of HUVEC colocalized with fibroblast cells in a Matrigel 3D coculture assay (Supplemental Figure 15). To examine whether Cth contributed to α-SMA–positive mesenchymal cell recruitment into tumor vasculature, we administered Ad-miR-*Cth* to mice after 7-day injection of Ex-3LLC or B16F10. Cth expression and H_2_S production were upregulated in the lungs of Slc7a5^iEC–KO^ mice, and administration of Ad-miR-*Cth* suppressed Cth protein expression as expected (Supplemental Figure 16–18). In Ex-3LLC–bearing mice, Ad-miR-*Cth* suppressed the number of PECAM1 or α-SMA–positive cells in Slc7a5^iEC–KO^ mice (Figure 5C). In B16F10-bearing mice, Ad-miR-*Cth* did not suppress PECAM1-positive area in control or Slc7a5^iEC–KO^ mice (Figure 5D), while the size of α-SMA–positive area were suppressed by Cth knockdown in Slc7a5^iEC–KO^ mice (Figure 5D). In both Ex-3LLC and B16F10-bearing mice, administration of Ad-*CTH* rescued the effect of Ad-miR-*Cth*, leading to restoration of α-SM–positive area in Slc7a5^iEC–KO^ mice (Figure 5, C and D). Collectively, these results suggest that increased production of H_2_S by Cth induction derived from LAT1 inhibition stimulated mesenchymal cell recruitment to the tumor vessel, which may lead to the formation of more mature, closer to the normal, blood vessels.

### Endothelial cell–specific LAT1 ablation improved drug delivery to tumor tissue mediated by vascular normalization.

**Figure 6:**
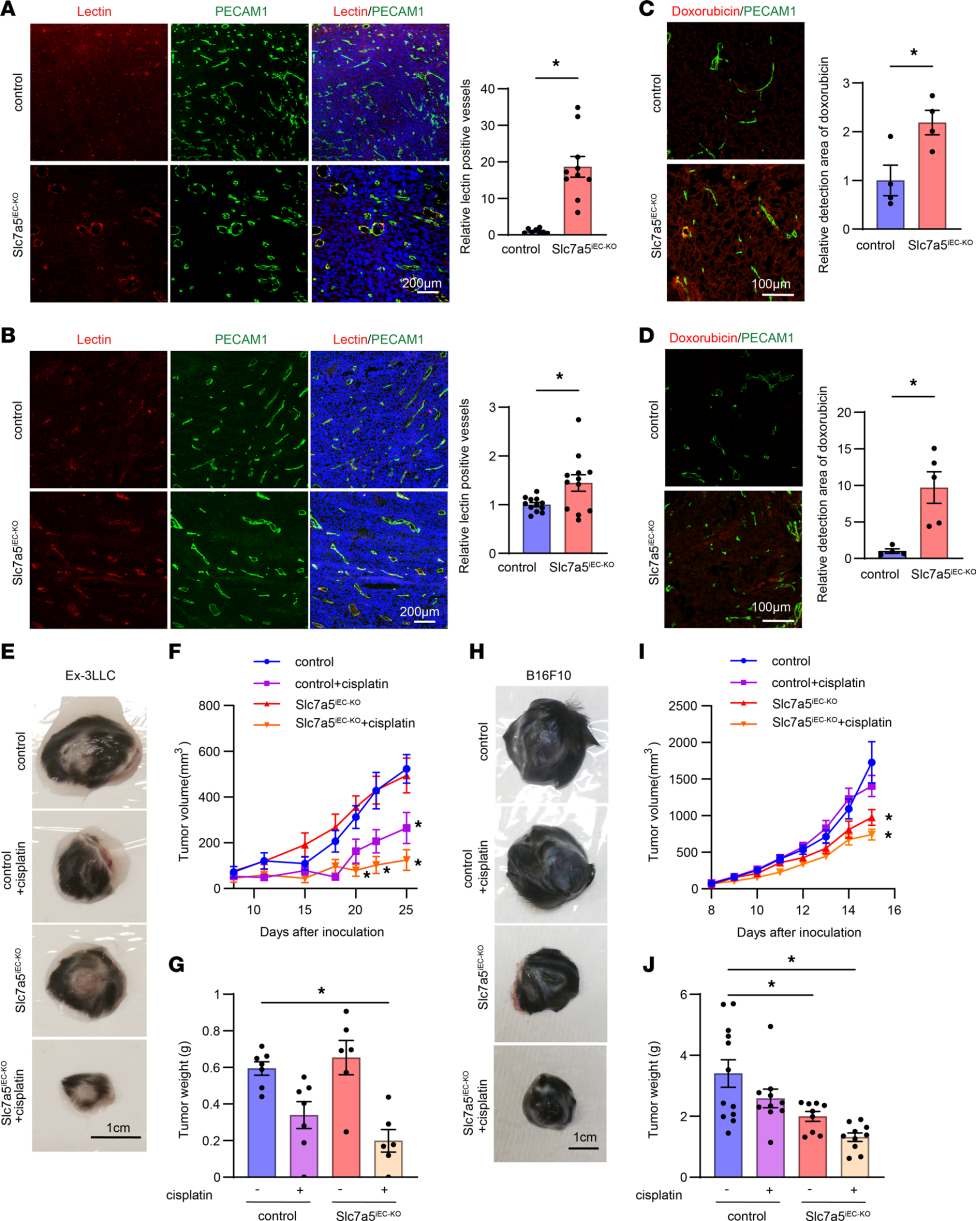
Endothelial cell–specific LAT1 ablation led to improved drug delivery to primary tumor mediated by vascular normalization. (**A**) Tumor vessel perfusion was examined in subcutaneous Ex-3LLC bearing mice by intravenous injection of FITC conjugated tomato lectin. The ratio (mean ± SEM, control; *n* = 8, Slc7a5^iEC–KO^ mice; *n* = 10) of lectin positive cells/PECAM1 positive cells were counted and normalized to that of control mice (the mean of the control mice was calculated as 1). (**B**) Tumor vessel perfusion was examined in subcutaneous B16F10 bearing control or Slc7a5^iEC–KO^ mice by intravenous injection of FITC-conjugated tomato lectin. Semiquantification of lectin positivity (mean ± SEM, control; *n* = 12, Slc7a5^iEC–KO^ mice; *n* = 12) was done as in **A**. (**C**) Tumor vessel perfusion was examined in subcutaneous Ex-3LLC–bearing mice by intravenous injection of doxorubicin. Fluorescent intensity of doxorubicin (mean ± SEM) was calculated from 4 randomly selected slices and normalized to that of control (= 1). (**D**) Tumor vessel perfusion was examined in subcutaneous B16F10-bearing control or Slc7a5^iEC–KO^ mice by intravenous injection of doxorubicin. Fluorescent intensity of doxorubicin (mean ± SEM) was calculated from 4 or 5 randomly selected slices and normalized to that of control (= 1). (**E**) Ex-3LLC solid tumor growth in control or Slc7a5^iEC–KO^ mice in the presence or absence of 10 mg/kg cisplatin at day 26. (**F**) Ex-3LLC tumor volume in each condition were estimated from the radius of the tumor. (mean ± SEM, control; *n* = 7, control + cisplatin; *n* = 7, Slc7a5^iEC–KO^ mice; *n* = 6, Slc7a5^iEC–KO^ + cisplatin; *n* = 6). (**G**) Ex-3LLC tumor weights at day 26 were shown. (**H**) B16F10 solid tumor growth in control or Slc7a5^iEC–KO^ mice in the presence or absence of 10 mg/kg cisplatin at day 15. (**I**) B16F10 tumor volume in each condition were estimated from the radius of the tumor. (mean ± SEM, control; *n* = 12, control + cisplatin; *n* = 10, Slc7a5^iEC–KO^ mice; *n* = 9, Slc7a5^iEC–KO^ + cisplatin; *n* = 10). (**J**) B16F10 tumor weights at 15 days were shown. (**A**–**D**) *P* values were determined by 2-tailed, unpaired *t* test. (**F**–**I**) *P* values were determined by 1-way ANOVA with Tukey-Kramer’s multiple comparisons test compared to control at each time point. *P < 0.05. Scale bars: 200 μm (**A** and **B**); 100 μm (**C** and **D**); 1 cm (**E** and **H**).

We also analyzed the perfusion of the primary tumor in control and Slc7a5^iEC–KO^ mice. When FITC-tomato lectin was intravenously injected into Ex-3LLC or B16F10-bearing mice, the number of endothelial cells stained with tomato lectin at the primary tumor injected sites was increased in Slc7a5^iEC–KO^ mice compared with that of control mice, indicating that endothelial cell–specific ablation of LAT1 improved the perfusion to the newly formed tumor vessels (Figure 6, A and B). Similarly, delivery of the anticancer drug doxorubicin to the primary tumor injection sites was improved in Slc7a5^iEC–KO^ mice compared with that of control mice (Figure 6, C and D). If this better perfusion is translated to improved delivery of chemotherapeutic drugs, normalization of tumor vasculature by LAT1 inhibition would carry therapeutic potential. To test this idea, cisplatin, an anticancer drug, was intraperitoneally administered to Slc7a5^iEC–KO^ mice and examined the Ex-3LLC or B16F10 tumor volume and size. In Ex-3LLC-bearing Slc7a5^iEC–KO^ mice, which exhibited an antimetastatic effect as seen in Figure 1C (Supplemental Figure 19), endothelial LAT1 inhibition augmented cisplatin-mediated tumor regression compared with control mice(Figure 6, E–G). In the case of B16F10-injected mice, cisplatin did not inhibit tumor growth in control mice, while tumor size apparently decreased in cisplatin-administered Slc7a5^iEC–KO^ mice (Figure 6, H–J). Taken together, these data suggest that vascular normalization caused by LAT1 inhibition might confer improved delivery of chemotherapeutic agents.

## Discussion

In agreement with Tărlungeanu et al. (15), we did not find gross vascular abnormalities in *Cdh5*-Cre–mediated LAT1 conditional knockout mice, which is reported to be more endothelial cell–specific than *Tie2*. Thus, LAT1 is likely to be dispensable for normal angiogenesis and maintenance of normal peripheral vasculature. Since Tărlungeanu et al. removed *Slc7a5* from Tie2-positive cells (i.e., endothelial and white blood cell progenitors) in a very early stage of vascular development and did not find a major vascular phenotype in their conditional knockout mice, LAT1 is also likely to be dispensable for physiological angiogenesis or vasculogenesis during development. In adult mice, cells in the blood brain barrier are the only endothelial cells positive for LAT1 (15). However, when tumors were transplanted into mice, some endothelial cells of tumor vasculature were found positive for LAT1 (Figure 1B, right panel). In 2 human cancer histological specimens, LAT1 was also detected in the endothelium of the tumor blood vessel (Figure 1A).

The fact that ablation of LAT1 from the endothelial cell did not affect the number of blood vessels around the tumor in the tumor-bearing mice (Figure 1, E and F) indicates that LAT1 is dispensable for tumor-induced angiogenesis as well as physiological angiogenesis discussed above. But removal of LAT1 from endothelial cells reduced the tumor growth at the injection sites and reduced the number and size of the lung metastases (Figure 1, C and D). Moreover, the shape of the blood vessel around the tumor appeared more normal looking in Slc7a5^iEC–KO^ mice (Figure 1, E and F). Taking these results together, we suspected that LAT1 in the endothelial cell may contribute to developing tumor type blood vessels both near tumor and metastatic sites, which are leakier than the normal blood vessels and allow tumor metastasis (24).

We utilized HUVEC for our in vitro analysis. To address the relevancy of HUVEC to the endothelial cell in the tumor vasculature, we confirmed that LAT1 inhibition led to VCAM1 suppression, and CTH induction seen in HUVEC was also observed in activated dermal or lung microvascular endothelial cells, which were derived from vasculature near primary or metastatic tumors, respectively (Supplemental Figure 20). Molecular mechanisms of tumor vessel normalization by LAT1 inhibition were investigated in HUVEC stimulated with inflammatory cytokines TNF-α or IL1-β, which have been reported to be present in the tumor microenvironment (25). In TNF-α or IL1-β–stimulated HUVEC, nanvuranlat inhibited the expression of VCAM1 and E-selectin leading to decreased monocyte attachment to the endothelium. Subsequently, we have shown that tryptophan depletion recapitulated administration of nanvuranlat. We also found that inhibition of MEK1/2-ERK1/2 restored VCAM1 upregulation in the presence of nanvuranlat. These findings were in stark contrast to multiple publications on the role of LAT1 in the cancer cell, which reported that LAT1 inhibition leads to leucine depletion, subsequently inhibiting mTOR activation and cancer cell proliferation (26). Regarding the role of mTOR signaling in VCAM1 induction, Wang et al. demonstrated that rapamycin suppressed VCAM1 by inhibiting mTORC2-dependent Akt signaling (27). In our hands, nanvuranlat did not affect Akt activation (Figure 2D). In addition, rapamycin treatment did not change the expression level of genes upregulated by nanvuranlat (Figure 3B). Thus, the molecular mechanism of VCAM1 suppression by LAT1 inhibition was likely to be different from that by rapamycin. Analyzing global gene expression changes in HUVEC in the presence or absence of nanvuranlat led us to identify another molecular target of LAT1 inhibition, CTH (Figure 3A). CTH upregulation was observed with tryptophan restriction and reversed by MEK1/2-ERK1/2 inhibition (Figure 3, B and C), suggesting that this enzyme is likely to be at least one of the target molecules downstream of tryptophan and MAPK. Given that CTH catalyzes a reaction that generates a gas mediator H_2_S from cystathionine, the observed effect induced by CTH upregulation is likely to be mediated by H_2_S. In fact, H_2_S overproduction by administering an H_2_S donor molecule in tumor-injected mouse models mimicked the effect of nanvuranlat (Figure 4, G and H). This CTH upregulation and increased H_2_S generation not only affected endothelial cell adhesion molecule expression but also modified the structure of the tumor related vasculature (Figure 5, B–D), at least partially, through recruiting mesenchymal cells around the vasculature. The change in tumor related blood vessels induced by endothelial cel–specific LAT1 ablation translated into better perfusion to the tumor (Figure 6, A–D), which could be exploited by delivering more chemotherapeutic reagents to the tumor, as we have shown in Figure 6. Although we have shown that LAT1 ablation or functional inhibition in the tumor-associated vascular endothelial cells led to decreased VCAM1 expression in the endothelial cells and increased recruitment of mesenchymal cells near the vasculature, both of which were at least partially mediated by increased production of H_2_S from the endothelial cells when LAT1 was inhibited. It is possible that downstream effector molecules other than H_2_S may exist. Moreover, elucidating the detailed mechanism of decreased leakiness of the vasculature after LAT1 inhibition in the tumor-related endothelial cells is another interesting question yet to be solved.

Targeting tumor vasculature was started with anti-VEGF therapy, which aimed to inhibit tumor angiogenesis (28, 29). However, antiangiogenesis therapy, especially as a monotherapy, had limited success (30). Subsequently, the usefulness of anti-VEGF reagents in combination with cytotoxic chemotherapy was demonstrated, and this increased efficacy was determined to derive from decreased leakiness of tumor blood vessels so that delivery of chemotherapeutic agents was improved (23). This changing view of anti-VEGF therapy seems to be parallel with the emergence of tumor vascular normalization as a major aim when targeting tumor vasculature. As shown above, LAT1 inhibition in endothelial cells had little, if any, effect on angiogenesis per se, but normalized tumor vasculature. These results were not consistent with previous reports that endothelial LAT1 inhibition suppresses angiogenesis (9, 10). We speculate that endothelial cells in other studies may be exposed to much stronger proangiogenic signal than our tumor injection models. To fully address this question, elucidation of how tumor microenvironment induces LAT1 in the tumor vasculature endothelium may be required.

Cantelmo et al. demonstrated tumor vascular normalization by inhibiting a glycolytic enzyme PFKFB3 (31). Endothelial cell–specific inhibition of the enzyme led to decreased expression of adhesion molecules, including VCAM1 and E-selectin, to less vascular permeability, and to increased pericyte coverage, all of which were very similar to what we observed in the tumor vasculature with endothelial cell–specific LAT1 ablation. They also showed that endothelial PFKFB3 inhibition improved the delivery and efficacy of chemotherapy. We found that some tumor-related endothelial cells started to express LAT1,while Cantelmo et al. demonstrated that tumor-related endothelial cells become more reliant on glycolysis. In both cases, inhibiting initial adaptation of endothelial cells, i.e., LAT1 expression (although we did not yet know what drives LAT1 expression) or metabolic shift to glycolysis to the tumor microenvironment, led to normalization of tumor-related blood vessels. Whether this concept is generalizable is an interesting question for future research. From the practical standpoint, because PFKFB3 is expressed ubiquitously and is indispensable for many cells outside of the tumor microenvironment, targeting LAT1, which has more limited expression pattern, may be more tolerable.

A clinical trial of nanvuranlat as an antitumor agent is ongoing in Japan. In light of our study demonstrating that LAT1 inhibition in the tumor vasculature decreased lung metastasis as well as primary tumor growth, patients with cancer may benefit from receiving nanvuranlat at an early stage.

In summary, LAT1 expression by the tumor-associated endothelial cells was likely to mediate abnormal characteristics of tumor vasculature and its inhibition may normalize tumor-associated vasculature rather than inhibiting angiogenesis per se. Thus, we believe that our study suggests that LAT1 inhibition can be a novel strategy for normalizing the tumor microenvironment in addition to its role for suppressing tumor proliferation.

## Methods

### Sex as a biological variable.

In our study, we examined both male and female mice in in vivo solid tumor and metastasis models because they exhibited the same phenotype in preliminary studies, and thus sex was not considered a biological variable.

### Materials.

Nanvuranlat, a selective LAT1 inhibitor, and Sulfobutylether-β-cyclodextrin (Captisol) were provided by J-Pharma. TNF-α and IL1-β recombinant proteins were purchased from PeproTech. Rapamycin, LY294002, PD98059, PD0325901, SB203580, SP600125, and H-89 were from WAKO. Bio-repository human colon or breast cancer tissue was purchased from Reprocell.

### Mice.

*Slc7a5*^flox/flox^ mice were generated by UNITECH according to the plan as summarized in Supplemental Figure 1. In short, the loxP sequence was inserted to upstream of the start codon in the first exon, and Kozak sequences (ccdccacc) were also inserted between the loxP sequence and start codon. FRT-Neo-FRT-loxP cassette were inserted to the first intron. Cre recombinase led to deletion of the first exon including coding and splice donor region and resulted in *Slc7a5* gene knockout attributed to avoiding normal splicing. PCR primer pairs for genotyping *Slc7a5*^flox/flox^ mice were indicated as follows: 5′-AGTTGGATGATGACCAGTAAGGTCT-3′ and 5′-TCCCTAAAGAGATTACCATCCATAAG-3′ for *Slc7a5*^flox/flox^. *Cdh5*-Cre-ERT2 mice were provided by Kubota (Keio University) (11). PCR primer pairs for genotyping *Cdh5*-Cre-ERT2 mice were indicated as follows: 5′-AGGTTCGTTCACTCATGGA-3′ and 5′-TCGACCAGTTTAGTTACCC-3′ for *Cdh5*-Cre-ERT2 mice. In tamoxifen administration, *Slc7a5*^flox/flox^; *Cdh5*-Cre-ERT2 mice weighing 15–25 g were intraperitoneally injected with 2.5 mg of tamoxifen for each mouse every day for 5 days. Mice were used for experiments after more than 1 week of the tamoxifen treatment.

### Cell culture.

HUVEC, human dermal microvascular endothelial cells (HMVEC), and human skin fibroblast cells were purchased from Clonetics. Human pulmonary microvascular endothelial cells (HPMEC) were purchased from PromoCell. The 293FT cell line was purchased from Thermo Fisher Scientific. HUVEC were cultured in EGM-2 (PromoCell) and other microvascular endothelial cells were cultured in EGM-MV2 (PromoCell). For amino acid starvation, endothelial cells were cultured in custom-made amino acid deprived MCDB131 medium (Nacalai), which was supplemented by amino acids as needed according to the composition of MCDB131 media, containing 5 ng/mL VEGF (Peprotech), 50 ng/mL basic FGF (Peprotech), 8 U/mL heparin (Sigma-Aldrich), 2% dialyzed FBS (Thermo Fisher Scientific), and 5 U/mL penicillin/streptomycin (Nacalai). For amino acid supplementation, 4.1 mg/L tryptophan, 130 mg/L leucine, 15 mg/L methionine, or 35 mg/L cysteine as a 1 × final concentration, which was calculated from amino acid concentrations of MCDB131, was added to EGM2 media. Human skin fibroblast, human embryonic kidney–293 (HEK-293) cells (ATCC CRL-1573), and B16F10 melanoma cells (ATCC CRL-6475) were cultured in DMEM (Sigma-Aldrich, D5796) supplemented with 10% heat-inactivated FBS and 5 U/mL penicillin/streptomycin. Lewis lung carcinoma–derived cell line (Ex-3LL; JCRB cell bank JCRB1716) and U937 human monocyte (U-937; ATCC CRL-1593.2) were cultured in RPMI 1640 medium (Nacalai, 30263-95) with 10% heat-inactivated FBS and 5 U/mL penicillin/streptomycin.

### Adenovirus vector construction.

The human CTH or miRNA expressing adenovirus vectors were constructed using GATEWAY cloning system (Thermo Fisher Scientific). Adenovirus titer was measured using Adeno-X Rapid Titer Kit (Clontech) according to the manufacturer’s instruction. The coding sequence of human *CTH* was cloned in the previous study (8). DNA sequences of miRNA were shown below.

miR-*CTH* (NM_001902; human *CTH*) forward: 5′-TGCTGAAGTGTGGCAGGAAACCTTGTGTTTTGGCCACTGACTGACAC AAGGTTCTGCCACACTT-3′, reverse: 5′-CCTGAAGTGTGGCAGAACCTTGTGTCAGTCAGTGGCCAAAACAC AAGGTTTCCTGCCACACTTC-3′; miR-*Cth* (NM_145953; mouse *Cth*) forward: 5′-TGCTGTTTCCAAGCAATTCCTTGTTGGTTTTGGCCACTGACTGACCAA CAAGGTTGCTTGGAAA-3′, reverse: 5′-CCTGTTTCCAAGCAACCTTGTTGGTCAGTCAGTGGCCAAAACCAA CAAGGAATTGCTTGGAAAC-3′.

### Solid tumor and lung colonization models.

For solid tumor model, 1 × 10^6^ Ex-3LLC or B16F10 cells were subcutaneously implanted into the flank region of *Slc7a5*^flox/flox^(control) or Slc7a5^iEC–KO^ mice. For cisplatin treatment, 10 mg/kg cisplatin (MARUKO) or PBS (vehicle) were intraperitoneally injected every week. For the lung colonization model, 2.5 × 10^5^ Ex-3LLC or B16F10 cells were intravenously injected to control or Slc7a5^iEC–KO^ mice. For GYY4137 administration, PBS (control) or 80 mg/kg GYY4137 was intraperitoneally injected to C57BL/6J mice 2days before tumor cell injection. For premetastatic niche formation, cancer cells were stained with Cell Tracker Red CMTPX Dye (Thermo Fisher Scientific, C34552) according to manufacturer’s instruction. For adenovirus administration, 1 × 10^8^ ifu Ad-EGFP (control), Ad-miR-*Cth* or/and Ad-*CTH* were intravenously injected to control or Slc7a5^iEC–KO^ mice after 7 days of tumor implantation. For lectin administration, 2.5 mg/kg FITC-conjugated *Lycopersicon esculentum* (tomato) lectin (Vector laboratories, FL-1171) was intravenously administered to mice 15 minutes before solid tumor removal. For doxorubicin administration, doxorubicin hydrochloride (1.5 mg/kg; Nippon Kayaku) was intravenously injected and tumors were collected after intracardiac perfusion with PBS, according to the previous study (16). Fluorescence intensity of FITC-lectin and doxorubicin was detected by FV3000 confocal fluorescent microscope (OLYMPUS).

### IHC.

Solid tumor and mouse tissue were fixed with 4% paraformaldehyde phosphate buffer solution (Nacalai) for 4 hours, washed with PBS for 1 hour, dehydrated in 70%–100% ethanol in 10% increments for 30 minutes each and treated with chloroform for 1 hour 3 times. The pretreated samples were embedded in paraffin. Paraffin-embedded slices were emersed in xylene, deparaffined in 100%–70% ethanol in 10% decrements for 30 minutes each, washed in PBS, autoclaved in HistoVT 1 (Nacalai), and treated with methanol containing 3% hydrogen peroxide for 20 minutes. Sections were permeabilized in 0.05 M TBS-T buffer, blocked in 0.05 M TBS-T buffer containing 5% goat serum, incubated in anti-PECAM1(Cell Signaling Technology, 77699S, 1:200), α-SMA (Cell Signaling Technology, 56856S, 1:200), or ki-67(Cell Signaling Technology, 12202S, 1:200) antibodies for 2 hours, and then treated with HRP-conjugated rabbit/mouse IgG antibodies (Nichirei Biosciences, 413341, 414341) and DAKO polymer reagents (DAKO) for 30 minutes. DAB detection was performed in DAB solution (3 mg DAB, 78.8 mg Tris-Cl buffer and 6.8 mg imidazole dissolved in 10 mL H_2_O) for 5 minutes, incubated until becoming brown in DAB solution with 1 μL 30% H_2_O_2_, and then counterstained in hematoxylin solution. Microscopic images were obtained using BZ-X700 Fluorescence Microscope (KEYENCE).

### Immunofluorescence.

Bio-repository human colon or breast cancer tissue was deparaffinized, permeabilized, and blocked as described in the IHC section. Slices were incubated in anti-PECAM1 (Cell Signaling Technology, 3528S, 1:200) and C-terminal fragments of human LAT1 (Transgenic, 1:200) antibodies for 2 hours and then subjected to DAB detection. Frozen slices were permeabilized in PBS containing 1% BSA and 0.3% Triton X-100 for 10 minutes on ice, blocked by PBS containing 1% BSA and incubated in anti-Pecam1 (BD Biosciences, 550274, 1:100), mouse Lat1 (Transgenic, 1:100), Vcam1 (BD biosciences, 553330, 1:100) or Cd11b (BD Biosciences, 557394, 1:100) at 4°C overnight. Sections were incubated in Alexa Fluor dye-conjugated IgG antibodies (Thermo Fisher Scientific, A-11006, A-11012, 1:400; 1:400) and DAPI (Thermo Fisher Scientific, 62248; 1μg/mL). Fluorescent detection was performed by FV3000 confocal fluorescent microscope (OLYMPUS).

### Quantitative real-time PCR.

Primary culture endothelial cells were treated with 100 μM captisol, nanvuranlat, GYY4137, 1 × 10^7^ ifu/mL Ad-EGFP (control) or Ad-miR-*CTH* for 48 hours before 4 hours of 10 ng/mL TNF-α or 20 ng/mL IL1β stimulation. Total RNA was extracted using ISOGEN (NIPPON GENE) from primary culture endothelial cells and reverse transcribed to cDNA with ReverTra Ace (TOYOBO). Realtime PCR was conducted using SYBR Green Real-Time PCR Master Mixes (Applied Biosystems) according to the manufacture’s instruction. Gene expression levels were normalized to cyclophilin A expression at each sample. All data were representative of 3 independent experiments and shown as mean ± SD (*n* = 3). DNA sequences of PCR primer pairs were summarized below: 5′-TCGCCACCTACCTGCTCAAG-3′ and 5′-GCCTTCACGCTGTAGCAGTTC-3′ for SLC7A5, 5′-TGGTTCCCAGTTTTTCATCTGC-3′ and 5′-CCATGGCCTCCACAATATTCA-3′ for Cyclophilin A, 5′-CATGAGTTGGTGAAGCGTCAG-3′ and 5′-AGCTCTCGGCCAGAGTAAATA-3′ for CTH, 5′- CATGGAATTCGAACCCAAACA-3′and 5′-TTTCGGAGCAGGAAAGCCCTGG-3′ for VCAM1, 5′-ATGAGCACACCTCACCAAAC-3′ and 5′-AGTTCTCCTGTGAGCAGGGTT-3′ for E-selectin, 5′-AGCTGTTTGAGAACACCTCGGCC-3′ and 5′-AGACTGGGAACAGCCCGTCCA-3′ for ICAM1, 5′-CTGCACGCCCTCTATGACA-3′ and 5′-TAAAAGGCAGCCAATCCTTCT-3′ for ASNS, 5′-GGTCCATTACCAGCTTTTGTACG-3′ and 5′-AATGTAGCGTCCAAATGCCAG-3′ for SLC7A11, and 5′-TGTTTGCTCTGGTGTTAGGAGT-3′ and 5′-CGCCTCGTTGAGGGAATTGAA-3′ for SLC1A4.

### Western blot analysis.

For whole cell lysate preparation, cells or mouse lung tissue was washed with ice-cold PBS, collected with a cell scraper or tweezers, and lysed with RIPA buffer (50 mM Tris-Cl (pH7.5), 150 mM NaCl, 0.5% NP40, and 1 mM EDTA). For nuclear extraction, cells were treated using Nuclear Extract Kit (Active Motif) according to the manufacture’s instruction. Cell lysates or nuclear extracts were isolated by SDS-PAGE gel and transferred to nitro cellulose membrane (GE healthcare). The membrane was blocked by TBS-T (100 mM Tris-Cl (pH7.5), 150 mM NaCl, 0.05% Tween20) containing 2.5% skim milk for 1 hour, incubated with anti-VCAM1 (R&D systems, BBA5, 1:500), CTH (Cell Signaling Technology, 19689S for human/mouse protein, 1:1000), Cth (proteintech; 12217-1-AP for mouse protein, 1:1000), phosphorylated-Akt (S433) (Cell Signaling Technology, 4060P, 1:1000), Akt (Cell Signaling Technology, 4691P, 1:1000), phosphorylated-ERK1/2 (T202/Y204) (Cell Signaling Technology, 4370T, 1:1000), ERK1/2 (Cell Signaling Technology, 9102S, 1:1,000), phosphorylated-p70S6K(T389) (Cell Signaling Technology, 9206S, 1:1,000), p70S6K (Cell Signaling Technology, 34475S, 1:1,000), phosphorylated-p65(S536) (R&D systems, MAB72261, 1:1,000), p65(R&D systems, MAB5078, 1:1,000), or β-actin (Sigma-Aldrich, A5441, 1:3,000) at 4°C overnight and treated with HRP-conjugated rabbit/mouse IgG antibodies (Cell Signaling Technology, 7076S, 7074S, 1:3,000) at room temperature for 1 hour. Chemiluminescence-based HRP immunodetection by SuperSignal West Dura Extended Duration Substrate (Thermo Fisher Scientific) was analyzed by ImageQuant LAS4000 (GE healthcare). All images shown in each figure were representative of 3 independent experiments.

### Quantification of intracellular amino acid by LC-MS/MS analysis.

Quantification of intracellular amino acid was performed using aTRAQ kit (AB Sciex) according to the manufacture’s instruction. Briefly, HUVEC were cultured in a 100 mm plate for 48 hours and then treated with 100 μM captisol or nanvuranlat in EGM2 media. After 0.5, 24, or 48 hours of nanvuranlat treatment, cells were washed with 10 mL of 5% mannitol 2 times and incubated with 1 mL ethanol containing 10 μL of sulfosalicylic acid containing norleucine for 10 minutes at room temperature. The supernatants were mixed with 500 μL of water. 400 μL of the mixture was added to 400 μL of CHCl_3_ and then centrifuged at 21,000*g* at 4°C for 15 minutes. The upper layer of the supernatant was passed through a 5 kDa-cutoff filter and centrifuged at 10,000*g* at 4°C for 2 hours. The filtrates were lyophilized until dry, followed by derivatization with the reagents supplied in the aTRAQ Kit for Amino Acid Analysis. The samples were labeled with isobaric tags that have distinguishable report ions to internal standards. aTRAQ reagent Δ8-labeled amino acid samples were mixed with the internal standards prelabeled with aTRAQ reagent Δ0 according to the manufacturer’s instructions. The 2 aTRAQ reagents are identical except for the number of isotopes they contain. The mixture was introduced into a tandem mass spectrometer QRAP6500 (AB Sciex) using a liquid chromatography Prominence UFLCXR system (Shimadzu). Labeled amino acids were chromatographically separated on AAA C18 Column (AB Sciex) with reverse-phase mode. Separated amino acids were detected using a tandem mass spectrometer in multiple-reaction monitoring mode. The amounts of amino acids were calculated by comparing the peak areas of amino acids between specimens and internal standards at a 1-to-1 ratio. As a quality control parameter, nonphysiological amino acids, norleucine and norvaline, added to sulfosalicyclic acid and borate labeling buffer, respectively, were used to assess the extraction and labeling efficiencies of the assay. All data were shown as mean ± SEM from 4 independent experiments.

### Monocyte adhesion assay.

HUVEC were seeded on a 24-well plate and cultured for 48 hours in EGM2 or amino acid supplemented/restricted media supplemented with 100 μM captisol, nanvuranlat, GYY4137, 1 × 10^7^ ifu/mL Ad-EGFP (control), or Ad-miR-*CTH* as needed. After the pretreatment, HUVEC were stimulated with 10 ng/mL TNF-α for 6 hours and overlaid with Calcein AM-labeled U937 monocyte for 1 hour. Cells were washed with Hanks’ buffered salt solution (Nacalai) and detected by FV3000 confocal fluorescent microscopy (OLYMPUS).

### DNA microarray analysis.

Total RNA was collected using ISOGEN (NIPPON GENE) from HUVEC, which were treated with 100 μM captisol or nanvuranlat for 48 hours and subjected to U133 plus 2.0 DNA microarray (Affymetrix). cRNA preparation and probe array hybridization were conducted according to the manufacture’s instruction. Gene ontology (GO) analysis was performed by DAVID (https://david.ncifcrf.gov/)

### H_2_S detection assay.

For detection of H_2_S production from primary culture endothelial cells, HUVEC were cultured in EGM2 with 100 μM captisol or nanvuranlat or in amino acid–supplemented or -restricted media for 48 hours, and then loaded with 5 μM HSip-1 DA(DOJINDO), an H_2_S fluorescent probe, for 30 minutes. HSip-1 fluorescence was detected by FV3000 confocal fluorescent microscope (OLYMPUS). For detection of H_2_S production from mouse lung tissue were removed from mice perfused with PBS containing 10 U/mL heparin (Sigma-Aldrich), cut into 40–60mg pieces, and dissolved in 1 mL lysis buffer (6 mol/L Urea, 2% SDS, 150 mmol/L Tris-HCl (pH 7.4)). Lysates were mixed with 160 μL of 100 μM HSip-1 working solution (DOJINDO) and incubated for 30 minutes at room temperature. HSip-1 fluorescence was detected by microplate reader SH-9000Lab (CORONA ELECTRIC) and converted into values per weight of pieces.

### Aortic ring assay.

After the tamoxifen treatment, the descending thoracic aorta of tamoxifen-injected *Slc7a5*^flox/flox^ (control) or Slc7a5^iEC–KO^ mice was removed, cut into 1 mm pieces, and embedded in growth factor reduced Matrigel (Corning). After 1 week of incubation with DMEM supplemented with 10% FBS, the aortic pieces were fixed with 4% paraformaldehyde phosphate buffer solution (Nacalai) for 10 minutes at room temperature and then stained with 3 U/mL rhodamine phalloidin (Thermo Fisher Scientific, R415) for 10 minutes. After washing with PBS, neovascularization from aortic pieces were observed by confocal fluorescent microscopy (OLYMPUS). The neovessel area was calculated using ImageJ software.

### Matrigel plug assay.

After the tamoxifen treatment, growth factor–reduced Matrigel (Corning) was subcutaneously implanted into the flank region of *Slc7a5*^flox/flox^(control), Slc7a5^iEC–KO^ or C57BL/6J mice. For GYY4137 treatment, 100 μM GYY4137 was premixed with the Matrigel before implantation. After 1 week, the Matrigel plug was removed, fixed with 4% paraformaldehyde for 1 hour, replaced with 10% to 30% sucrose solution in increments of 10%, and embedded into OCT compound (Sakura Finetek). Frozen sections were stained with anti-PECAM1(BD Biosciences, 550274, 1:100) and anti-α-SMA (Cell Signaling Technology, 56856S, 1:200) antibodies.

### Scratch migration assay.

Human fibroblast cells were cultured with 100 μM GYY4137 for 48 hours and then scratched with a P1000 pipet tip. After 24 hours, cells were labeled with 1 μM Calcein AM (DOJINDO) for 30 minutes and detected by FV3000 confocal fluorescent microscope (OLYMPUS). Migration area was calculated from fluorescent images by ImageJ software.

### Boyden chamber migration assay.

HUVEC were seeded on a 24-well plate and pretreated with 100 μM captisol or nanvuranlat in EGM2 media containing 1 × 10^7^ ifu/mL Ad-EGFP (control) or Ad-miR-*CTH* for 48 hours. After the pretreatment, 5 × 10^4^ Calcein AM-labeled human fibroblast cells were seeded on cell culture inserts with 8 μm pores (BD Biosciences). After 18 hours of coculture, fibroblast cells at the upper side of cell culture inserts were removed by a cell scraper. Fibroblast cells migrated to the bottom side on membrane were detected by FV3000 confocal fluorescent microscopy (OLYMPUS). Migration area was calculated by ImageJ software.

### Endothelium and fibroblast coculture tube formation assay.

HUVEC were seeded on a 24-well plate, cultured in EGM2 supplemented with 100 μM captisol or nanvuranlat for 48 hours, and labeled with 1 μM Calcein AM. Fibroblast cells were harvested, labeled with CellTracker Red CMTPX (Thermo Fisher Scientific), and mixed with 500 μL growth factor reduced Matrigel (Corning). The resulting mixture was overlayed on HUVEC and cultured in EGM2 supplemented with 100 μM captisol or nanvuranlat for 72 hours. Images were detected by FV3000 confocal fluorescent microscopy (OLYMPUS). The colocalization of HUVEC and fibroblast was calculated by ImageJ software.

### Statistics.

Data were analyzed by Excel and shown as the mean ± SD or SE as described in each figure legend or methods section. Statistical significance was tested by 2-tailed Student’s *t* test or ANOVA using GraphPad Prism10 (GraphPad Software). *P* < 0.05 was considered as significant.

### Study approval.

Animal experimental procedures were approved by the Experimental Animal Ethics Committee in Kyorin University (Permit Number: 92). All painful procedures were conducted under anesthesia, and all efforts were made to minimize suffering.

### Data availability.

Values for all data points are available in the Supporting Data Values file. All raw and processed expression data by microarray were available via NCBI Gene Expression Omnibus (GSE202790).

## Author contributions

JS, TK, HN, and HS conceived and designed the study. Methodology and/or data acquisition including providing animals were conducted by JS, TK, HN, TF, Y Kanki, YW, Y Kubota and NT. The original draft was written by JS and HS, and all coauthors reviewed the manuscript. Study supervision was carried out by HS.

## Supplementary Material

Supplemental data

Unedited blot and gel images

Supporting data values

## Version 1 08/20/2024

In-Press Preview

## Version 2 09/24/2024

Electronic publication
