# Supplementary material for: Endothelial cell–specific LAT1 ablation normalizes tumor vasculature
Source: JCI Insight. 2024 Aug 20;9(18):e171371. doi: 10.1172/jci.insight.171371 (PMC11457854; doi:10.1172/jci.insight.171371)
Supplement: Supplemental data [file jciinsight-9-171371-s130.pdf]

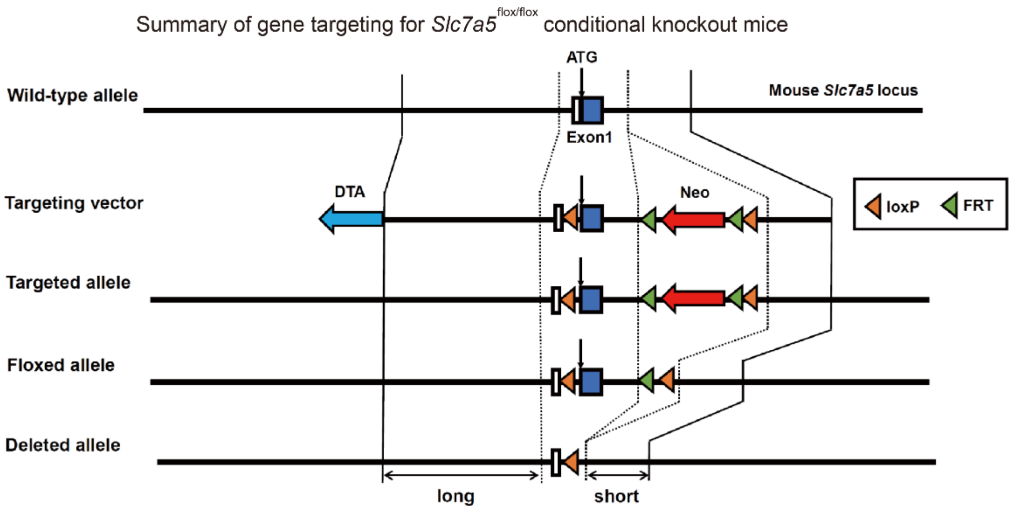

Supplemental Figure 1: Summary of gene targeting in Slc7a5$^{flox/flox}$ conditional knockout mice. A loxP sequence was inserted to upstream of the start codon in the first exon and FRT-Neo-FRT-loxP cassette were inserted to in the first intron.

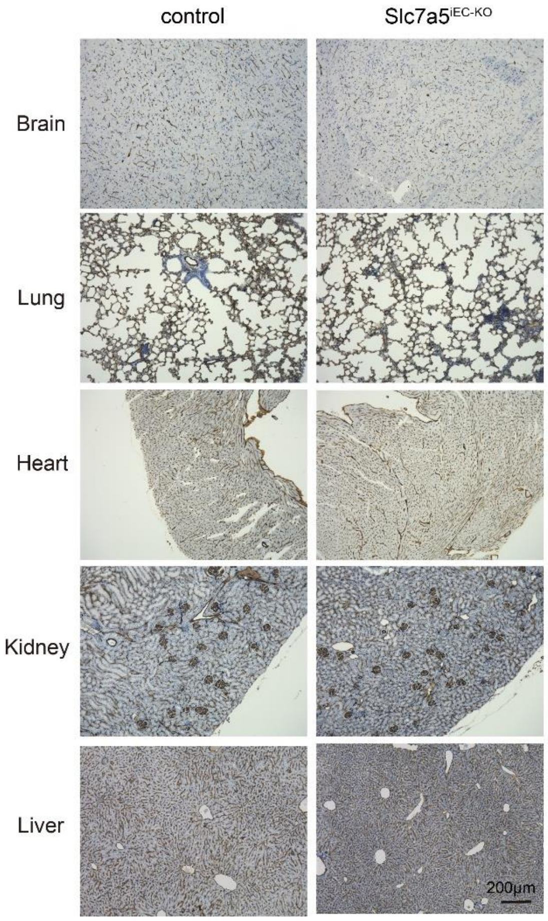

Supplemental Figure 2: Shape of vasculatures in control or Slc7a5^{iEC-KO} mice. Representative images of DAB staining using PECAM1 antibodies in brain, lung, heart, kidney, or liver in the absence of tumor.

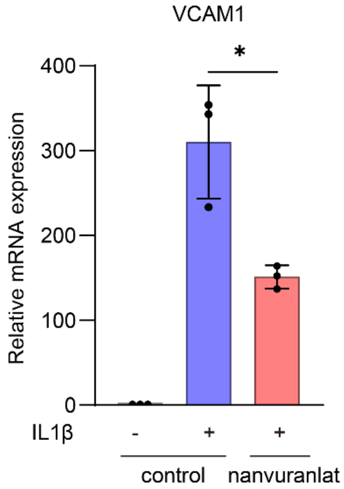

Supplemental Figure 3: VCAM1 mRNA expression in IL1β-stimulated HUVEC in the presence or absence of nanvuranlat. HUVEC was treated with 100µM Captisol or nanvuranlat for 48 hours, followed by stimulation with 20ng/mL IL1β for 4 hours. Data was shown as mean ± SD. P values were determined by 1-way ANOVA with Tukey's multiple comparisons test. *: $P<0.05$.

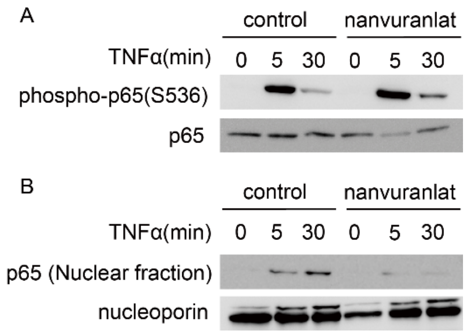

Supplemental Figure 4: Western blot analysis of NFκB p65 phosphorylation and nuclear localization in TNFα-stimulated HUVEC in the treatment with 100µM control or nanvuranlat. (A) Phosphorylation (S536) of p65 in TNFα-treated HUVEC. HUVEC was stimulated by 10ng/mL TNFα for 0, 5 or 30 minutes in the presence of 100µM Captisol or nanvuranlat. Whole cell lysates were subjected to western blot analysis. p65 pan antibody was used as a loading control. **(B)** Nuclear translocation of p65 in TNFα-treated HUVEC. HUVEC was stimulated by 10ng/mL TNFα for 0, 5 or 30 minutes in the presence of 100µM Captisol or nanvuranlat. Nuclear extracts from cells were subjected to western blot analysis. Nucleoporin was used as a loading control. Blots shown together were set up in parallel at the same time.

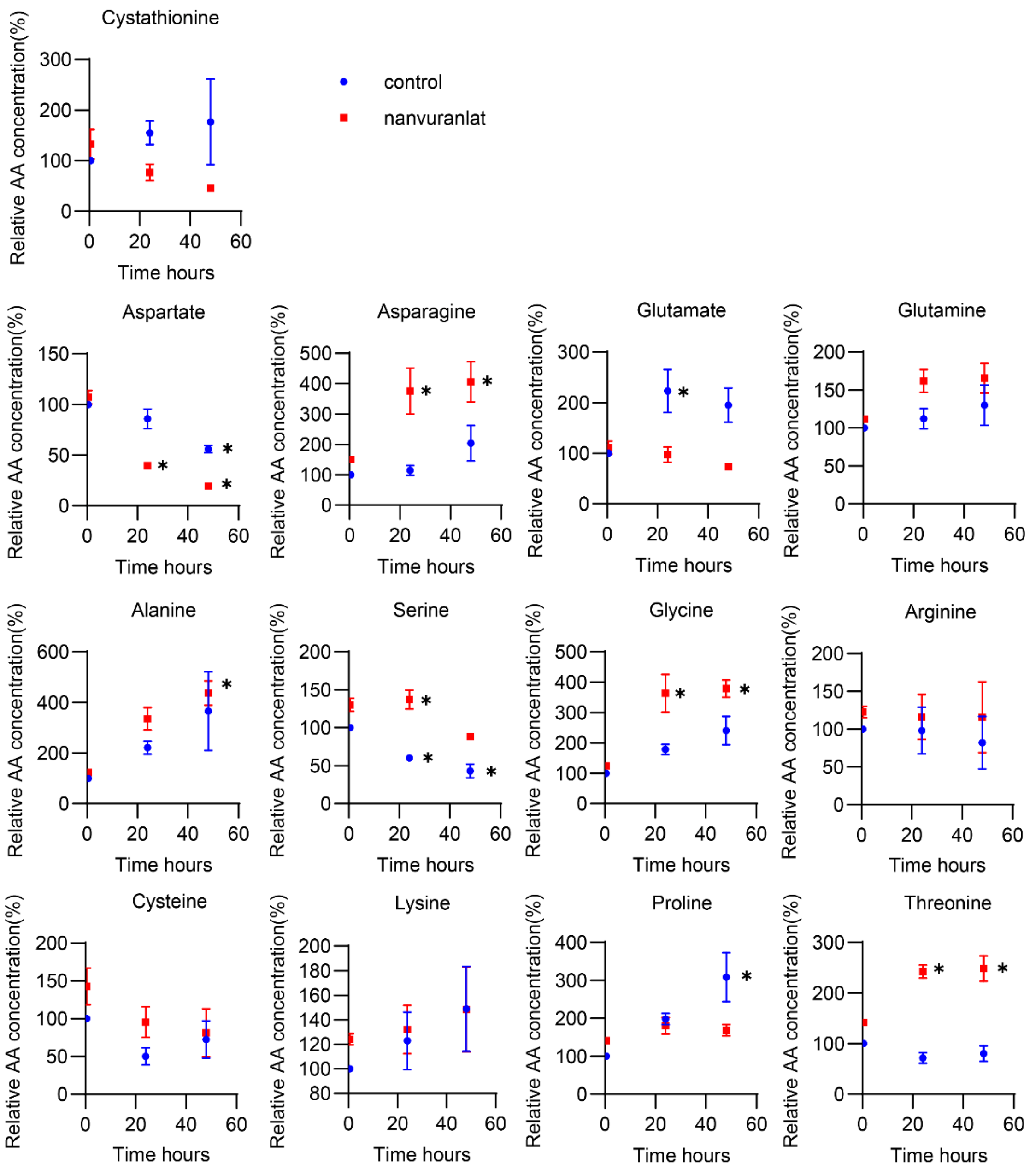

Supplemental Figure 5: Intracellular amino acid concentration by LC-MS/MS analysis in nanvuranlat-treated HUVEC. Plot graphs showed relative intracellular amino acid concentration in the presence of 100µM Captisol (blue circle) or nanvuranlat (red square). Data was shown as mean ± SE from 4 independent experiments. P values were determined by 1-way ANOVA with Tukey's multiple comparisons test compared to control at 0.5h.*: $p<0.05$,

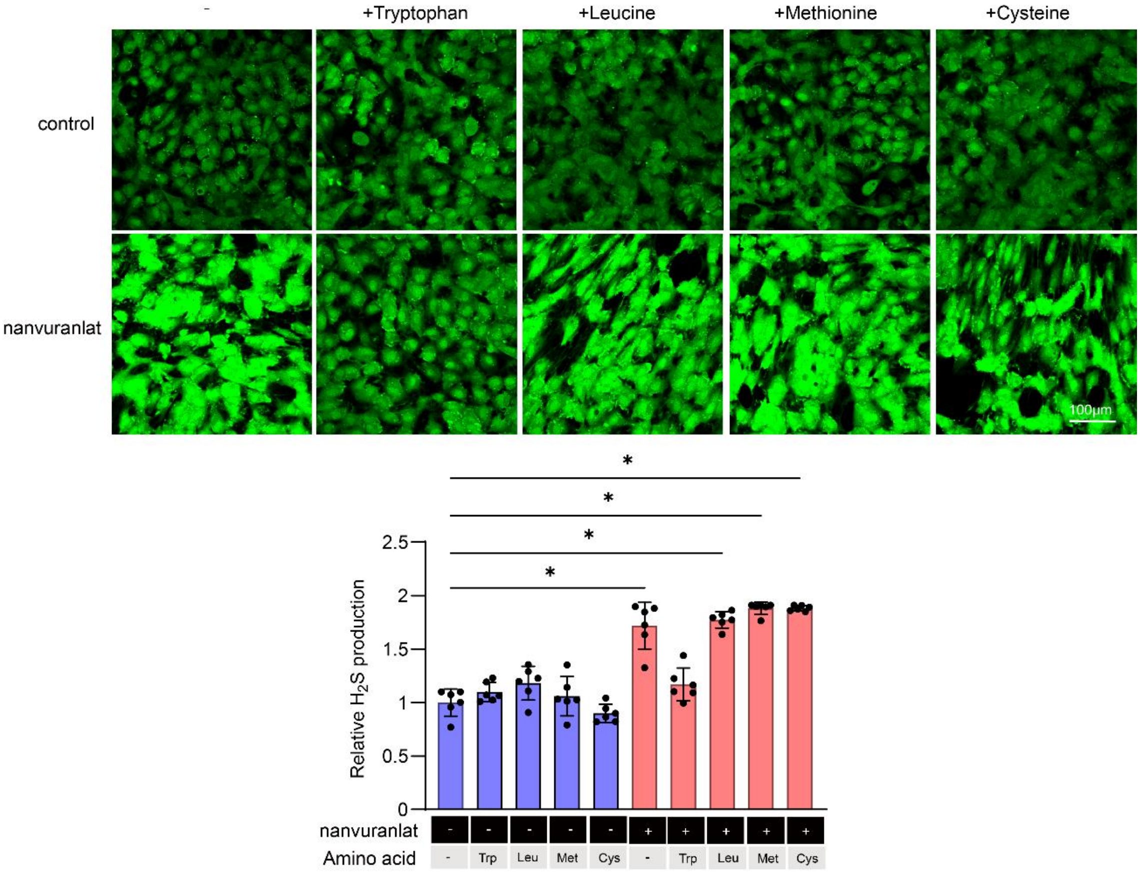

Supplemental Figure 6: H_2S production in HUVEC cultured in control or nanvuranlat supplemented with amino acids. HUVEC were loaded with HSip1-DA to visualize H_2S production. Bar graph indicated mean ± SD from 6 randomly selected images. P values were determined by 1-way ANOVA with Tukey's multiple comparisons test. *: $P<0.05$,

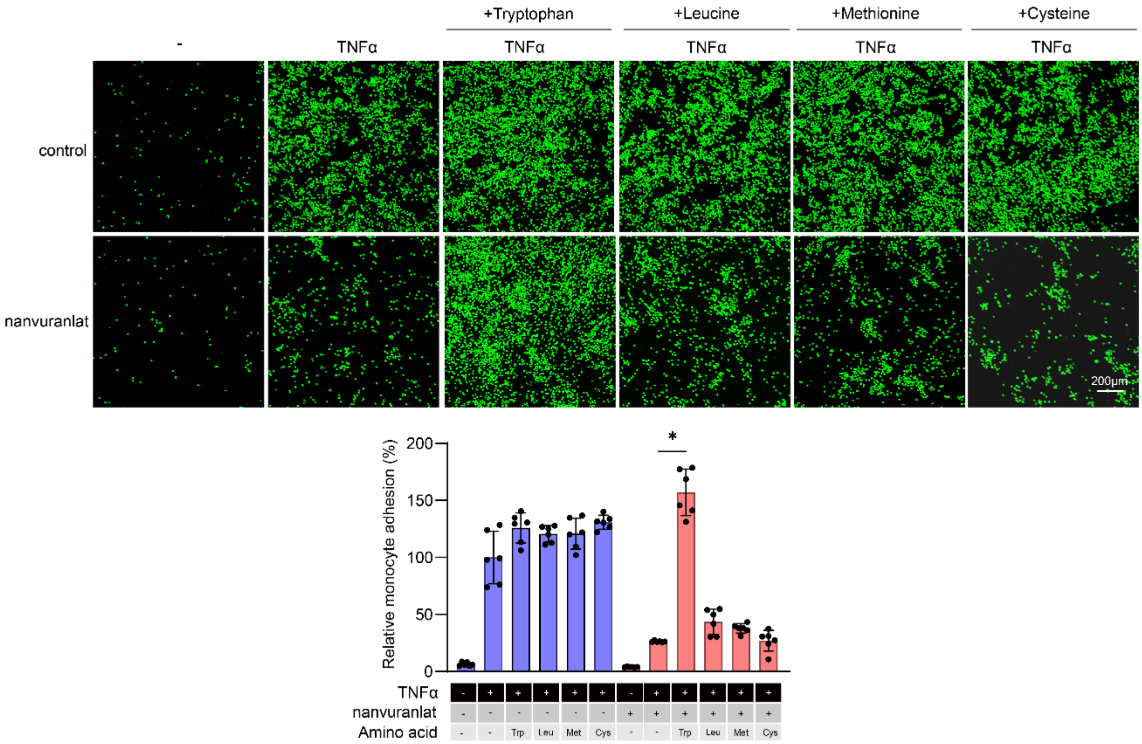

Supplemental Figure 7: Monocyte adhesion on TNFα-stimulated HUVEC cultured in control or nanvuranlat supplemented with amino acids. HUVEC were cultured for 48 hours in media containing 100μM Captisol or nanvuranlat supplemented with amino acids before TNFα stimulation. Bar graph indicated mean ± SD from 6 randomly selected images. P values were determined by 1-way ANOVA with Tukey's multiple comparisons test. *: $P<0.05$.

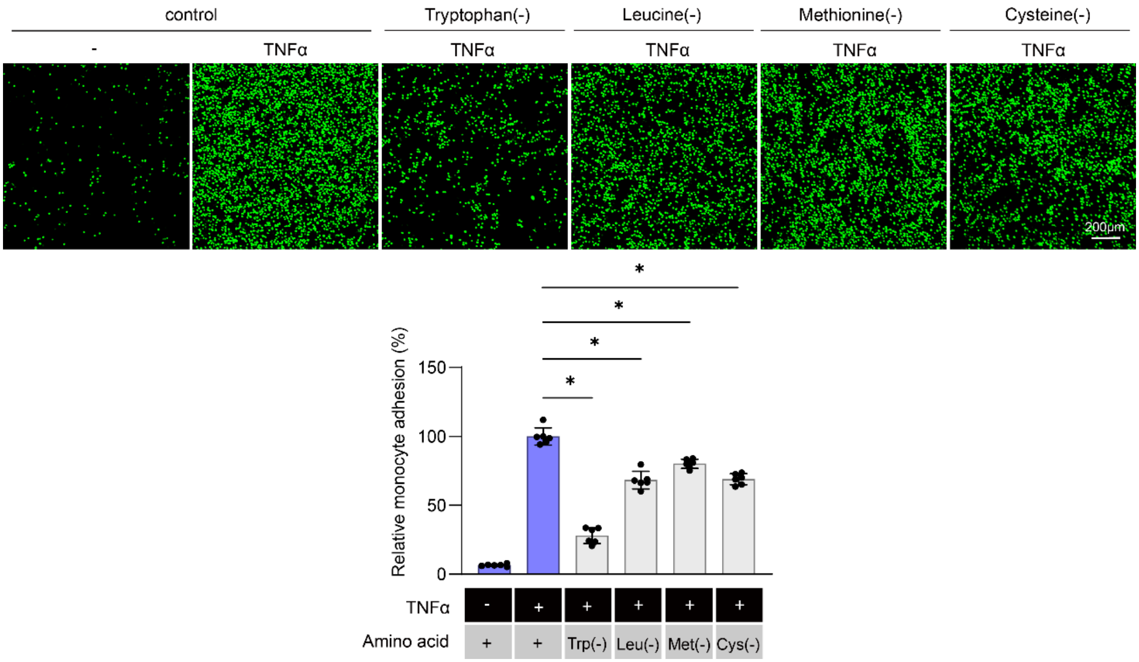

Supplemental Figure 8: Monocyte adhesion on TNFα-stimulated HUVEC cultured in amino acid depleted media. HUVEC were cultured for 48 hours in amino acid depleted media before TNFα stimulation. Bar graph indicated mean ± SD from 6 randomly selected images. P values were determined by 1-way ANOVA with Tukey's multiple comparisons test. *: $P<0.05$.

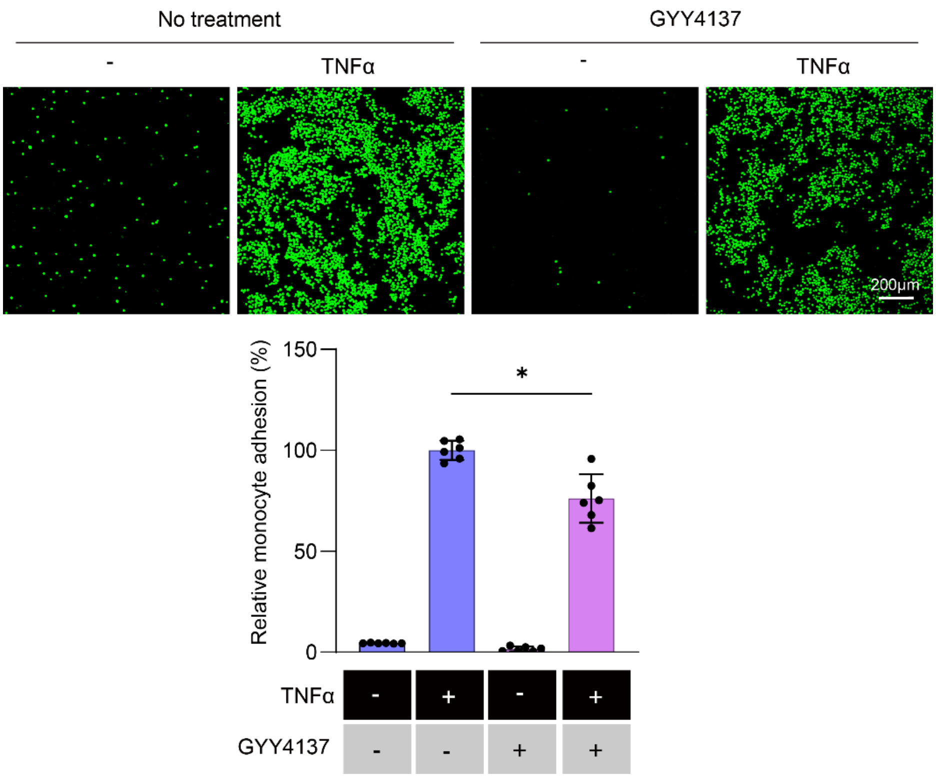

Supplemental Figure 9: Monocyte adhesion on TNFα-stimulated HUVEC treated with GYY4137. HUVEC were cultured in media containing 100μM GYY4137 before TNFα stimulation. Bar graph indicated mean ± SD from 6 randomly selected images. P values were determined by 1-way ANOVA with Tukey's multiple comparisons test. *: P<0.05.

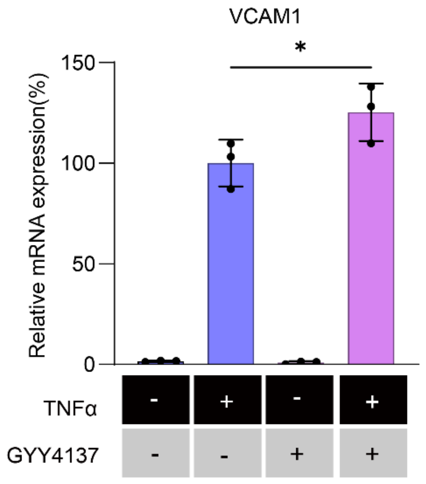

Supplemental Figure 10: VCAM1 mRNA expression in TNFα-stimulated HUVEC in the presence or absence of GYY4137. HUVEC were cultured in media containing 100μM GYY4137 before TNFα treatment. P values were determined by 1-way ANOVA with Tukey's multiple comparisons test. *: P<0.05.

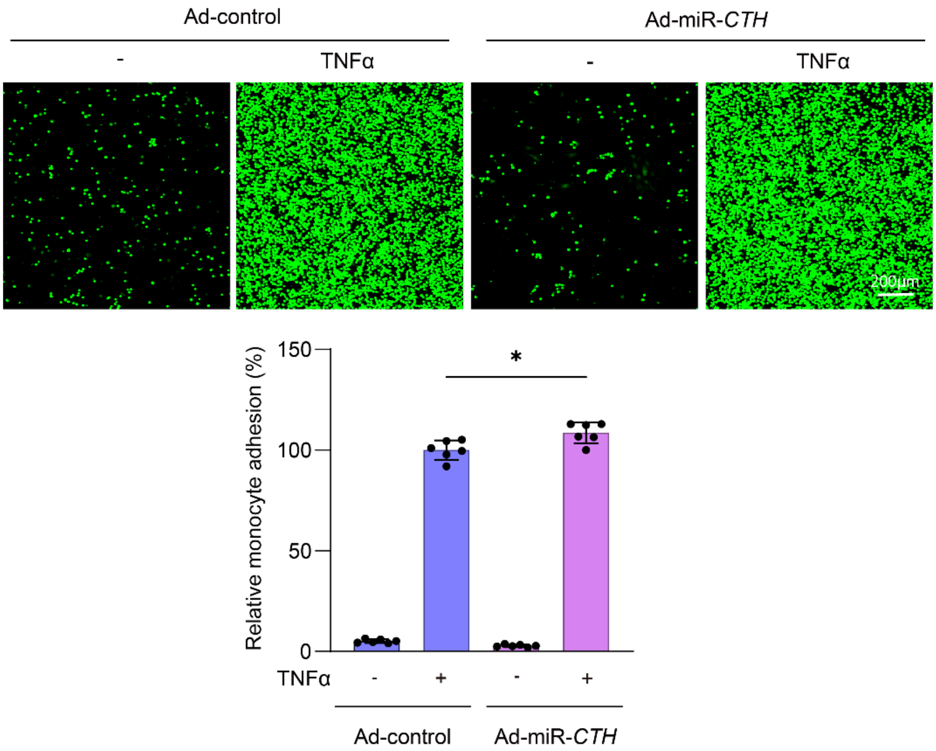

Supplemental Figure 11: Monocyte adhesion on TNFα-stimulated HUVEC treated with Ad-control or Ad-miR-*CTH*. HUVEC were cultured in media containing $1x10^7$ ifu/mL Ad-EGFP or Ad-miR-*CTH* for 48 hours before TNFα stimulation. Bar graph indicated mean ± SD from 6 randomly selected images. P values were determined by 1-way ANOVA with Tukey's multiple comparisons test. *: P<0.05.

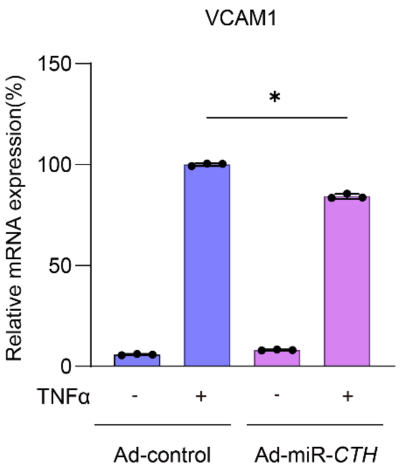

Supplemental Figure 12: VCAM1 mRNA expression in TNFα-stimulated HUVEC treated with Ad-control or Ad-miR-*CTH*. HUVEC were cultured in media containing 1×10^7 ifu/mL Ad-EGFP or Ad-miR-*CTH* for 48 hours before TNFα stimulation. Data was shown as mean ± SD. P values were determined by 1-way ANOVA with Tukey's multiple comparisons test. *: $P<0.05$.

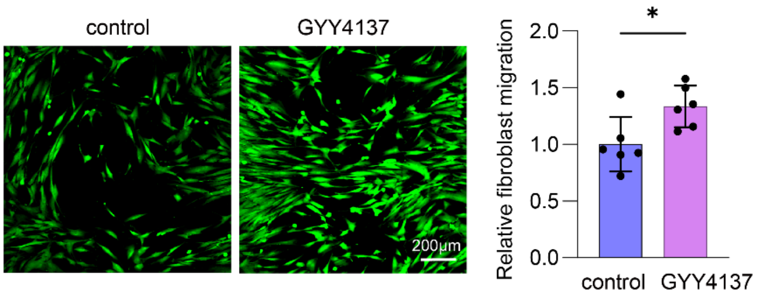

Supplemental Figure 13: Scratch migration assay in human fibroblast cells in the presence or absence of GYY4137. Human skin fibroblast cells were treated with 100µM GYY4137 for 48 hours. After 24 hours of scratch, cells were stained with Calcein AM solution and calculated by IMAGE J software to measure cell migration area. Data indicated mean ± SD from 6 slices. P values were determined by two-tailed unpaired t-test. *: P<0.05.

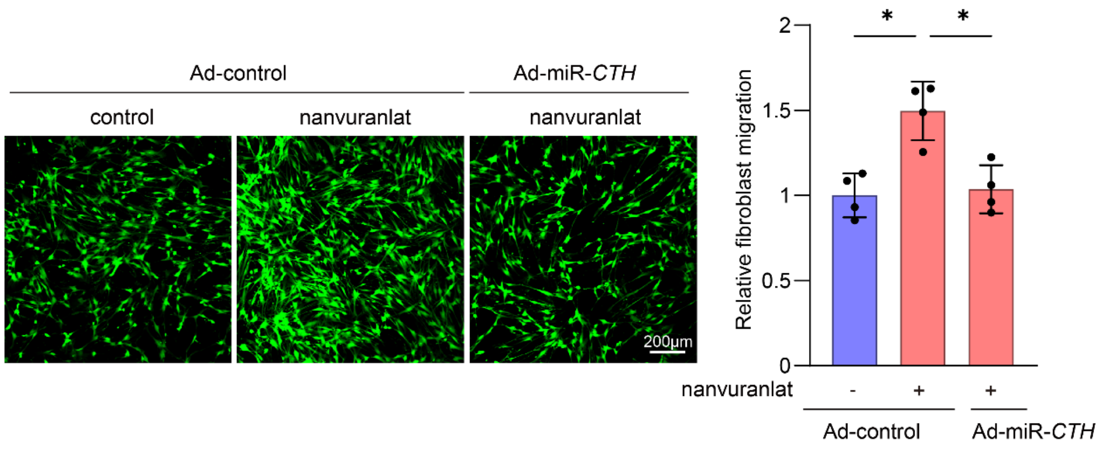

Supplemental Figure 14: Fibroblast migration assay in coculture with HUVEC using boyden chamber. HUVEC were treated with Ad-control or Ad-miR-*CTH*, followed by treatment with 100µM Captisol or nanvuranlat for 48 hours. Cell culture inserts with Calcein AM stained human fibroblast cells was set on the culture plates with HUVEC. After 24 hours, migrated fibroblast cells on bottom side of cell culture inserts were detected by fluorescent microscope and analyzed using IMAGE J software to calculate migrated cell area. Bar graph indicated mean ± SD from 4 randomly selected slices. P values were determined by 1-way ANOVA with Tukey's multiple comparisons test. *:P<0.05.

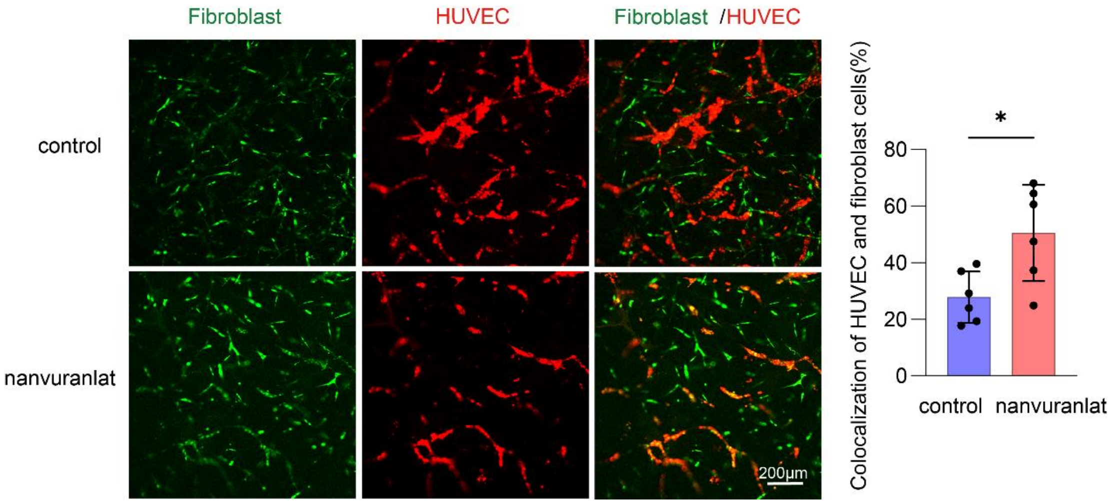

Supplemental Figure 15: 3D Matrigel co-culture tube formation assay using HUVEC and human fibroblast cells. HUVEC were stained with CellTracker Red CMTPX and cultured on growth factor reduced Matrigel. After 24 hours, Matrigel with Calcein AM labeled human fibroblasts was layered on HUVEC. Images were detected by fluorescent microscopy at 72 hours of coculture and were analyzed using IMAGE J to calculate colocalization area. Bar graph indicated mean ± SD from 6 randomly selected slices. P values were determined by two-tailed unpaired t-test. *: P<0.05.

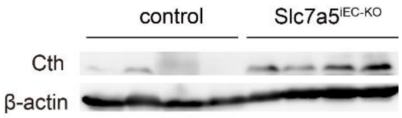

Supplemental Figure 16: Western blot analysis of Cth expression in the lung of control or Slc7a5^{iEC-KO} mice. Cth expression was shown in tissue extracts of lung of one mouse per lane. β-actin was used as a loading control. Blots provided together were set up in parallel at the same time.

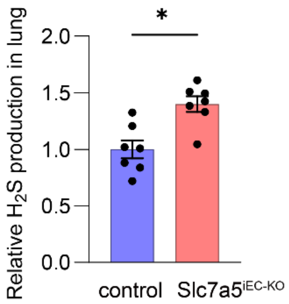

Supplemental Figure 17: H_2S production in the lung of control or Slc7a5$^{iEC\text{-}KO}$ mice. Tissue extracts of whole lung from 7 mice were subjected to fluorescent analysis using HSip-1. Data indicated mean ± SE. P values were determined by two-tailed unpaired t-test. *: $P<0.05$.

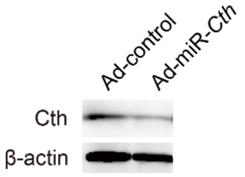

Supplemental Figure 18: Western blot analysis of Cth expression in the lung of C57BL/6 mice treated with Ad-control or Ad-miR-*Cth* intravenously. Representative data of 3 independent experiments was shown. β-actin was used as a loading control.

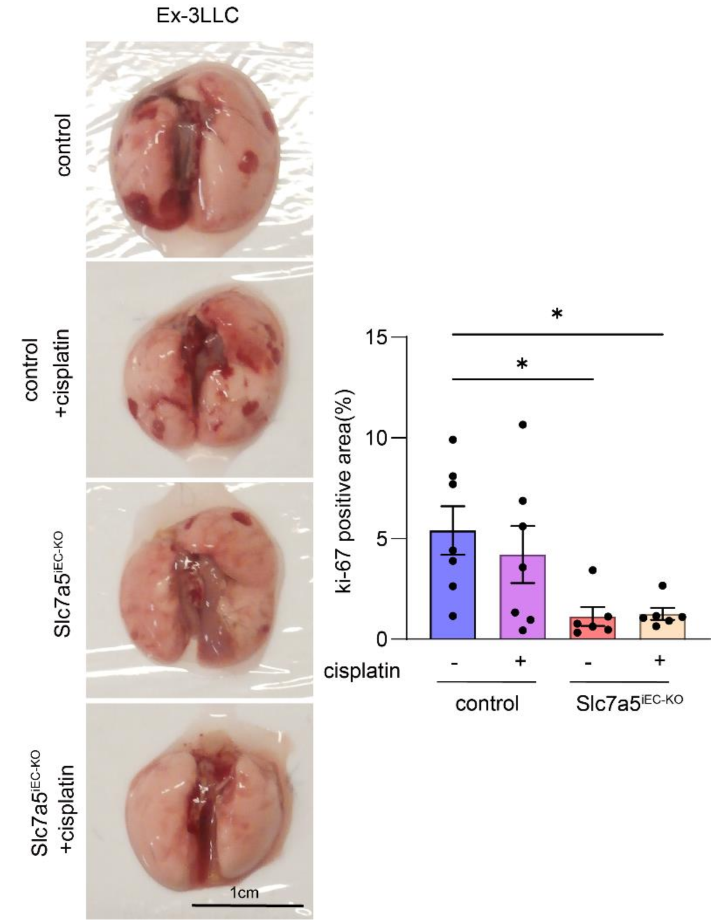

Supplemental Figure 19: Spontaneous metastasis in Ex-3LLC-bearing control or Slc7a5^{iEC-KO} mice treated with cisplatin. Macroscopic appearance of spontaneous lung metastasis was shown after 26 days of Ex-3LLC cell injection into mice in left panel. Bar graph showed the ki-67 staining area in lung of mice (mean ± SE, control; n=7, control + cisplatin; n=7, Slc7a5^{iEC-KO} mice; n=6, Slc7a5^{iEC-KO} +cisplatin; n=6). P values were determined by 1-way ANOVA with Tukey-Kramer's multiple comparisons test. *: $P<0.05$.

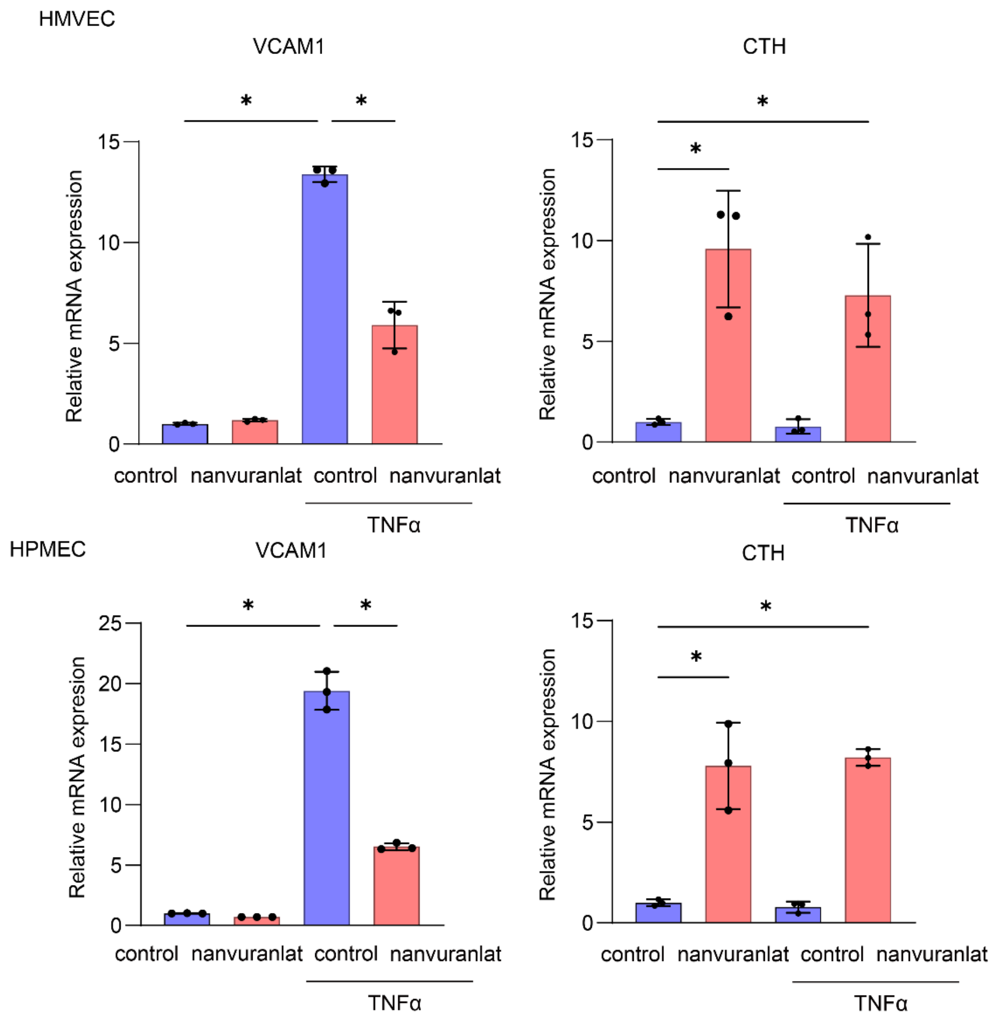

Supplemental Figure 20: VCAM1 or CTH expression in TNFα-treated endothelial cells derived from skin or lung. qPCR analysis was performed using total RNA from 10ng/mL TNFα-stimulated HMVEC and HPMEC in treatment with 100μM Captisol or nanvuranlat. P values were determined by 1-way ANOVA with Tukey's multiple comparisons test. *: P<0.05.
