# Supplementary material for: Endothelial cell–specific LAT1 ablation normalizes tumor vasculature
Source: JCI Insight. 2024 Aug 20;9(18):e171371. doi: 10.1172/jci.insight.171371 (PMC11457854; doi:10.1172/jci.insight.171371)

Full unedited gel for Figure 2B (VCAM1)

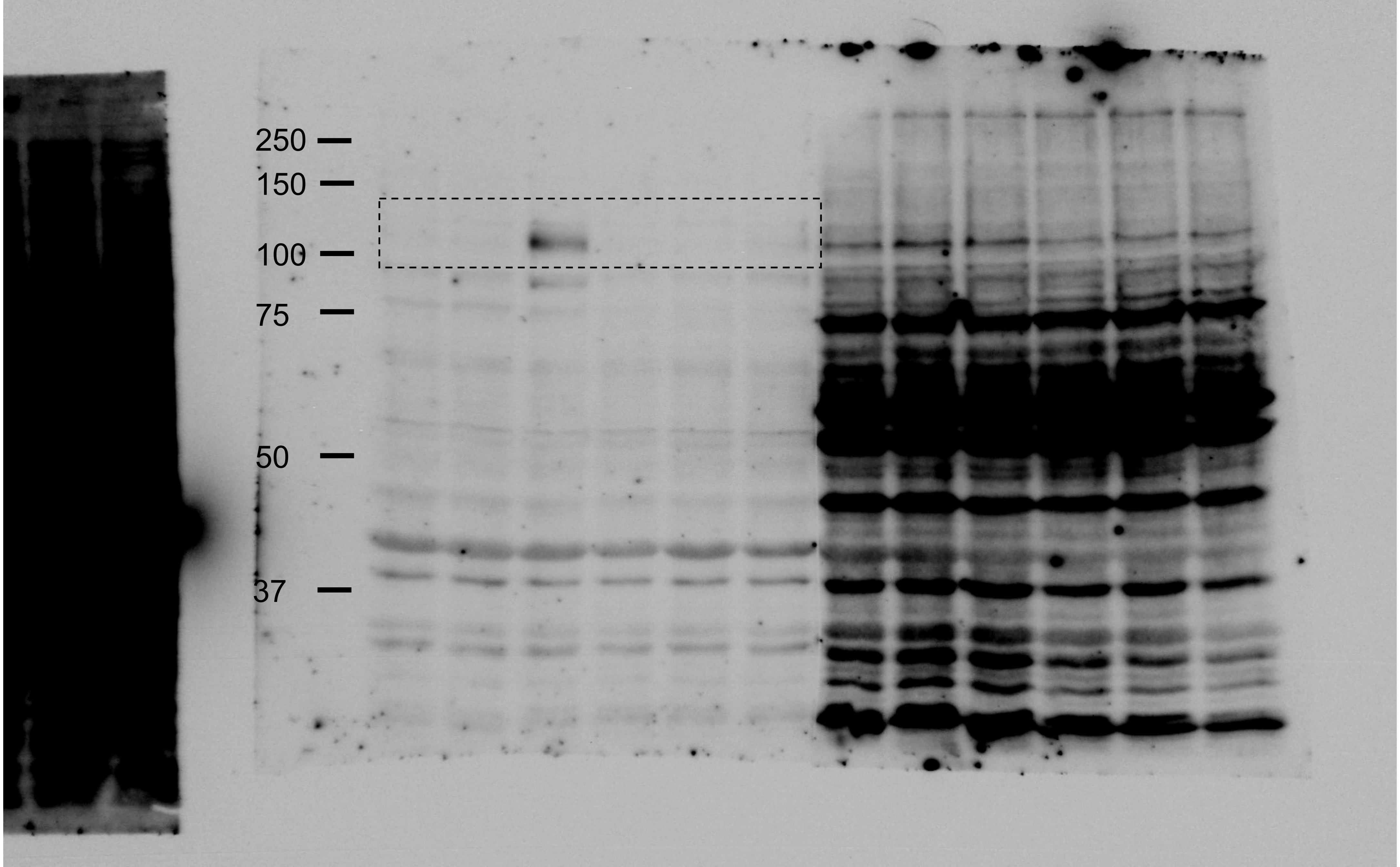

Full unedited gel for Figure 2B (β-actin)

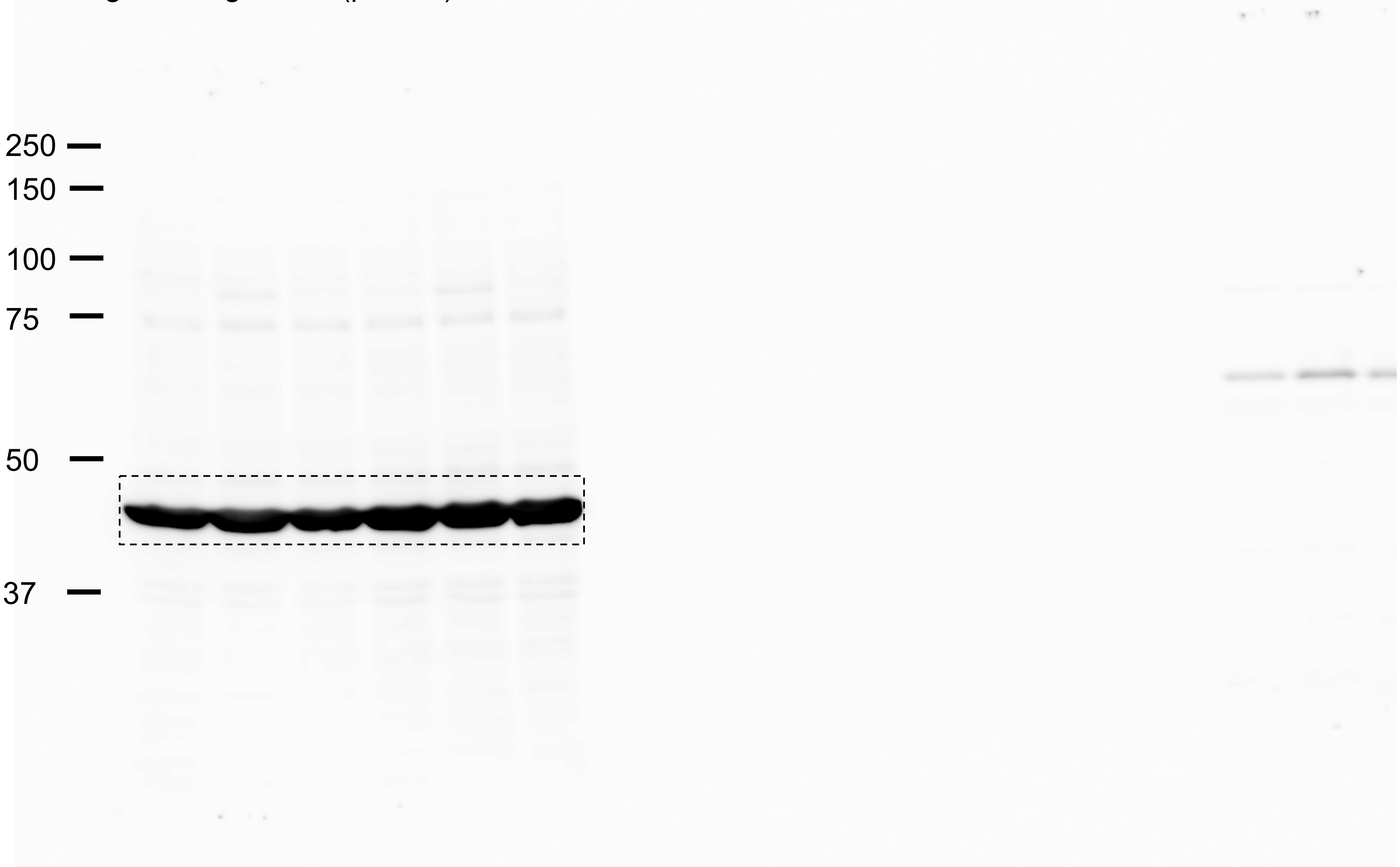

Full unedited gel for Figure 2D (Phospho-ERK1/2(T202/Y204))

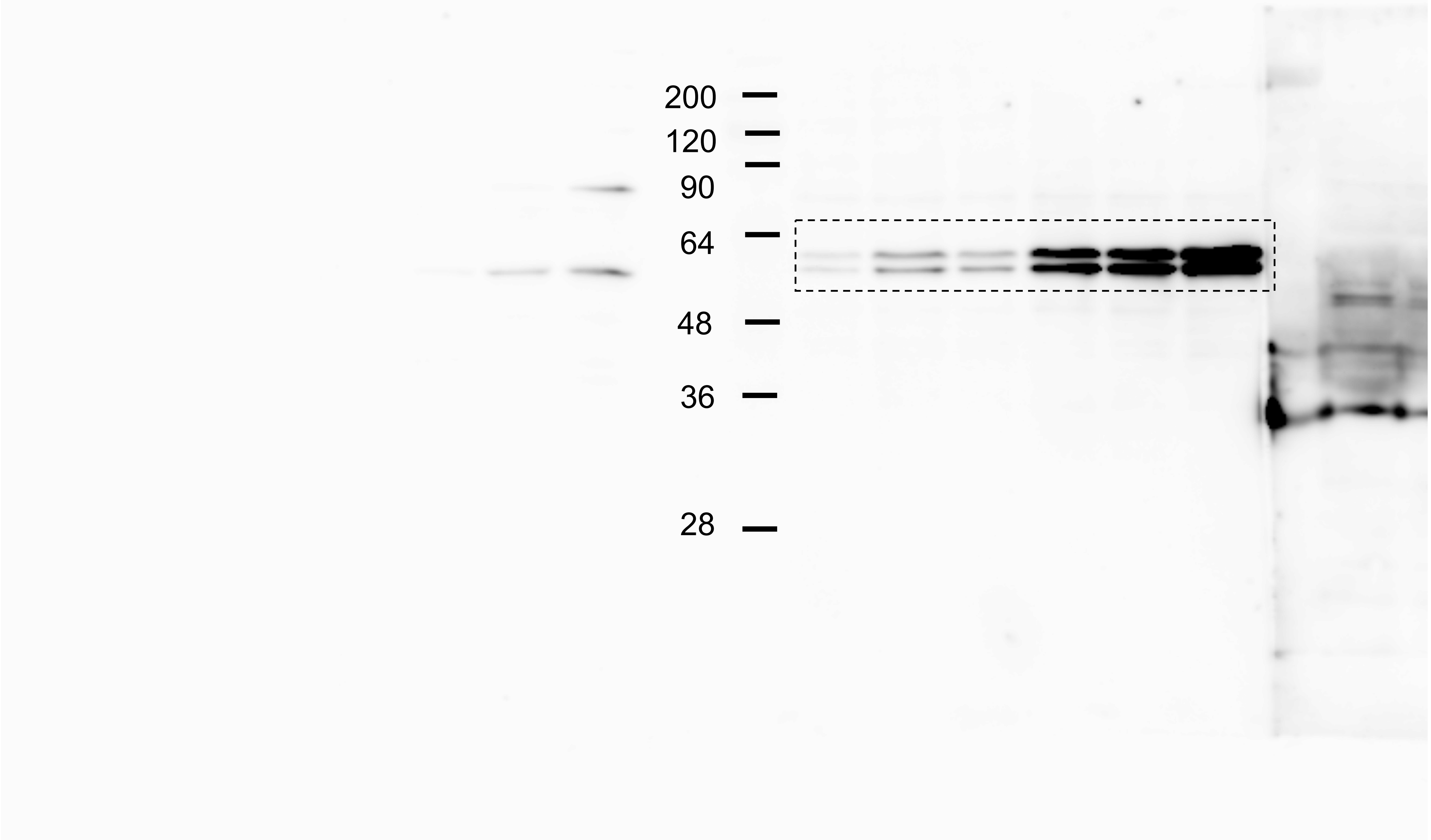

Full unedited gel for Figure 2D (ERK1/2)

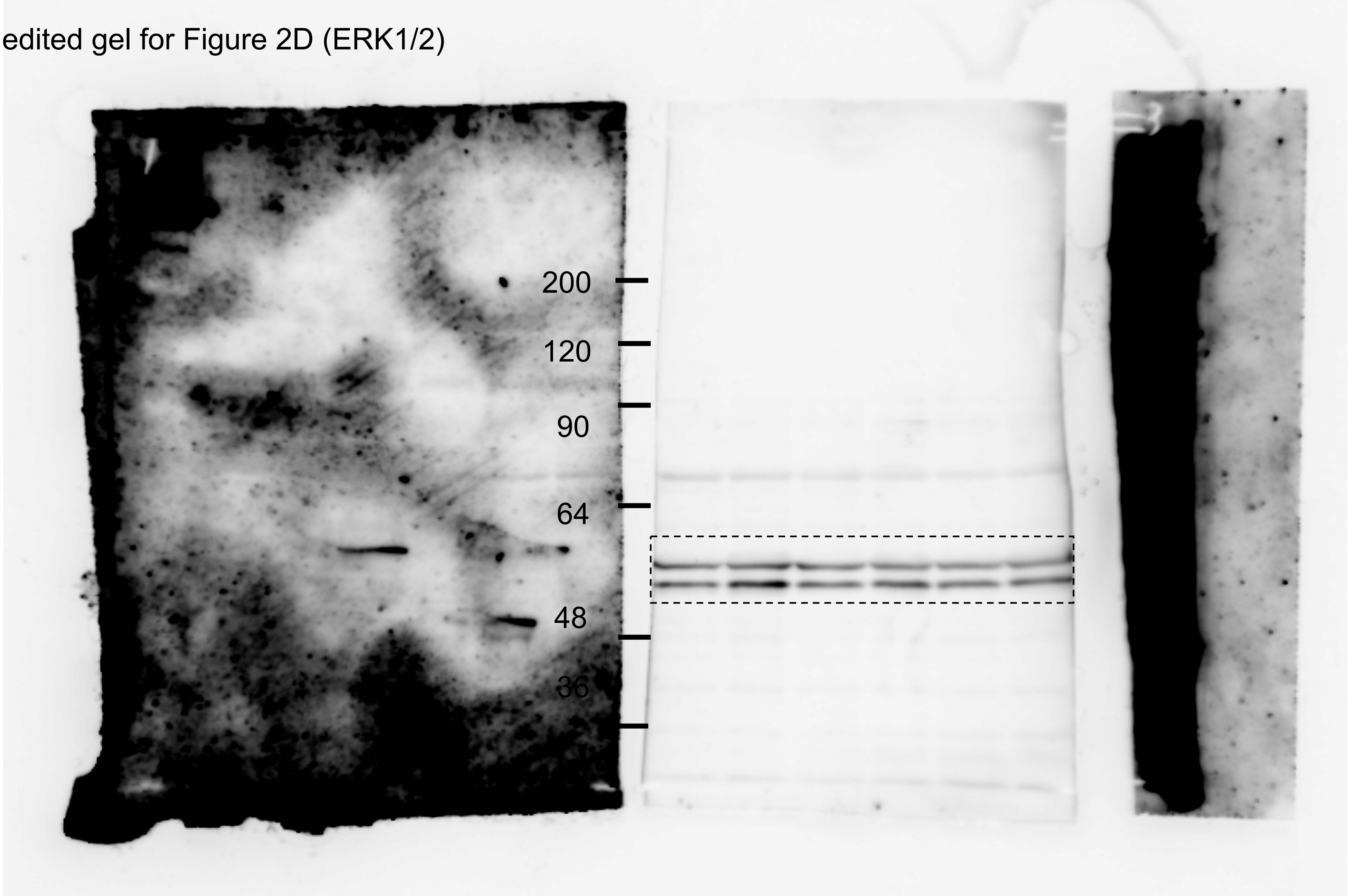

Full unedited gel for Figure 2D (Phospho-p70S6K(T389))

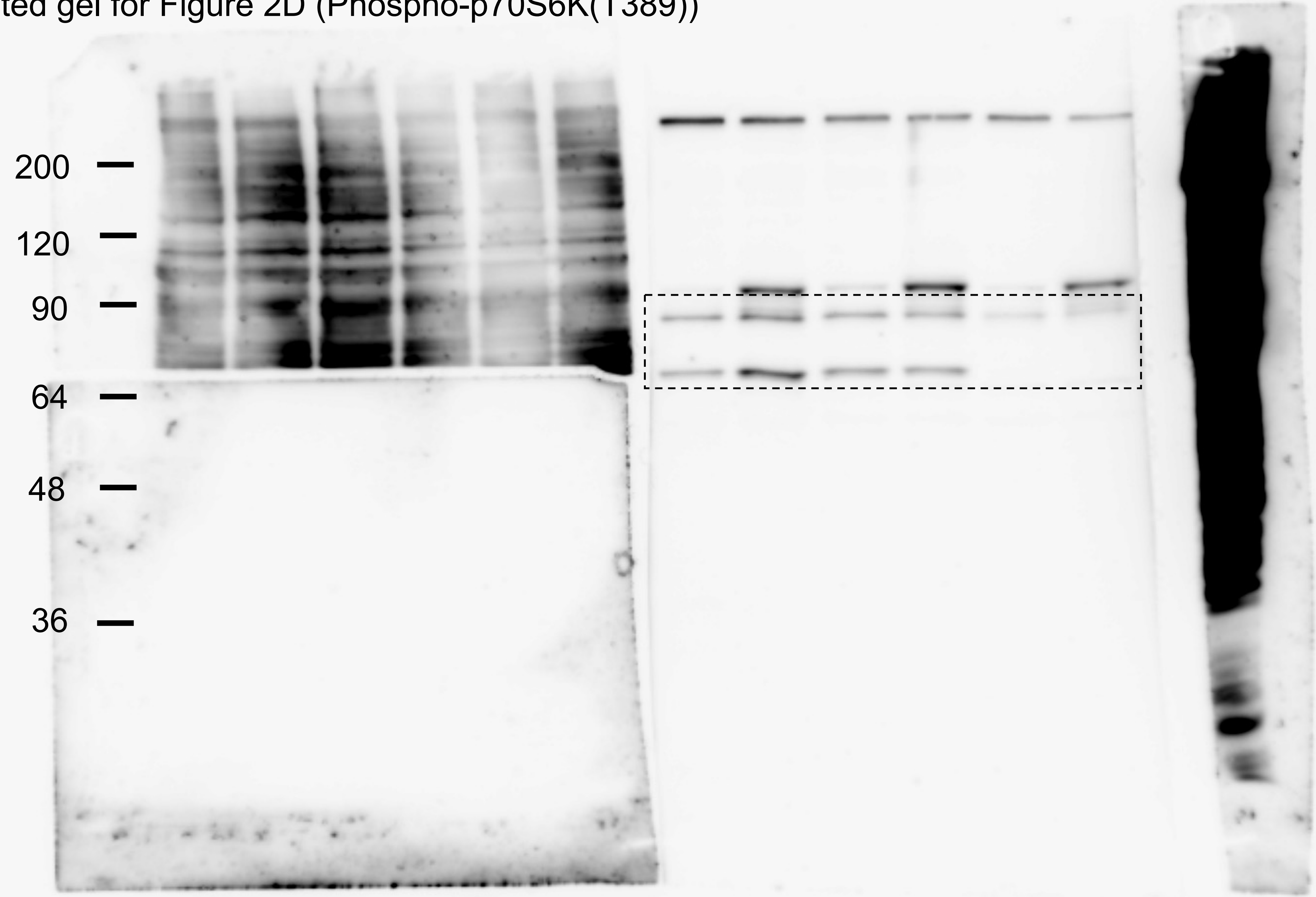

Full unedited gel for Figure 2D (p70S6K)

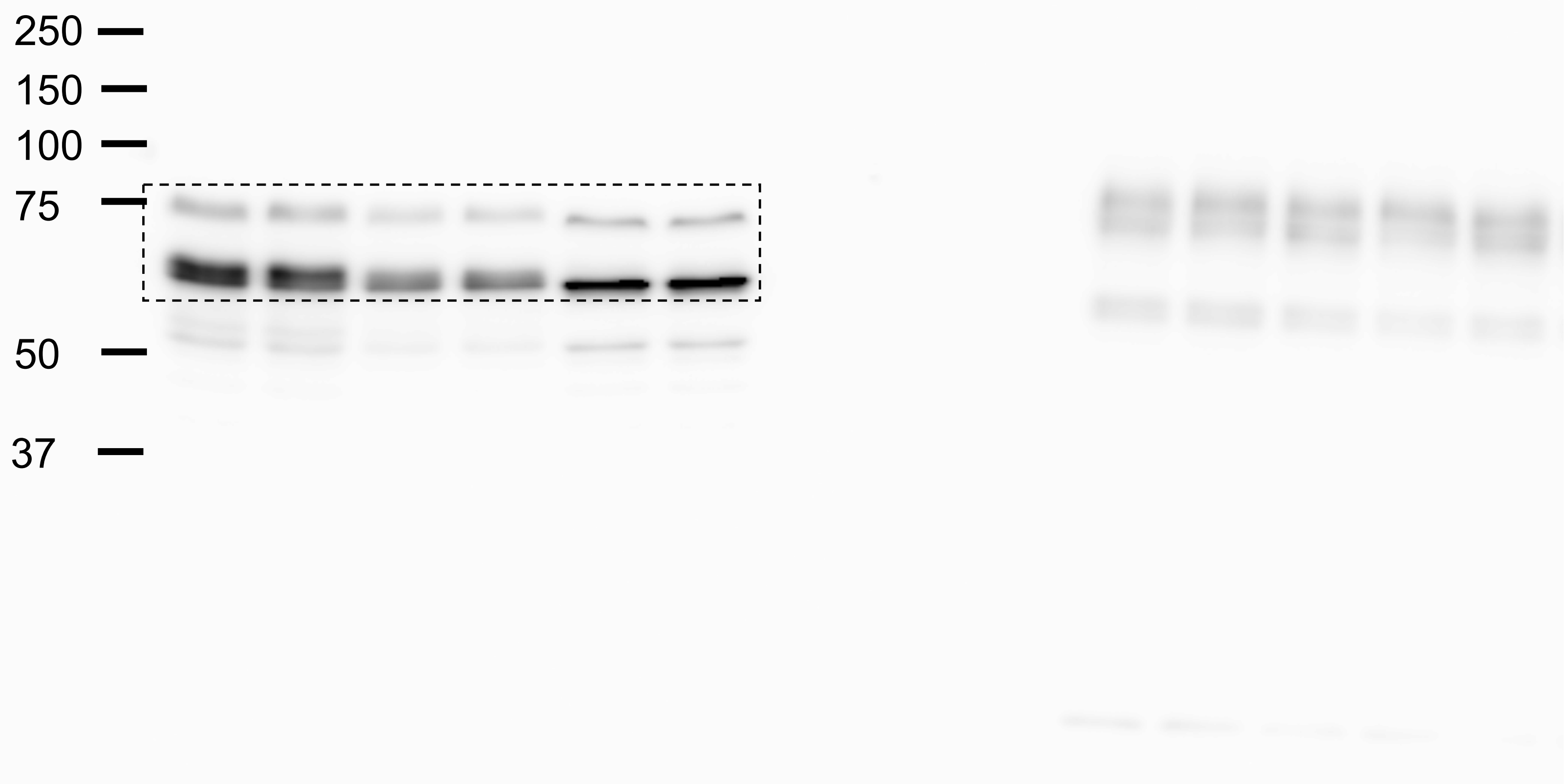

Full unedited gel for Figure 2D (Phospho-Akt(S473))

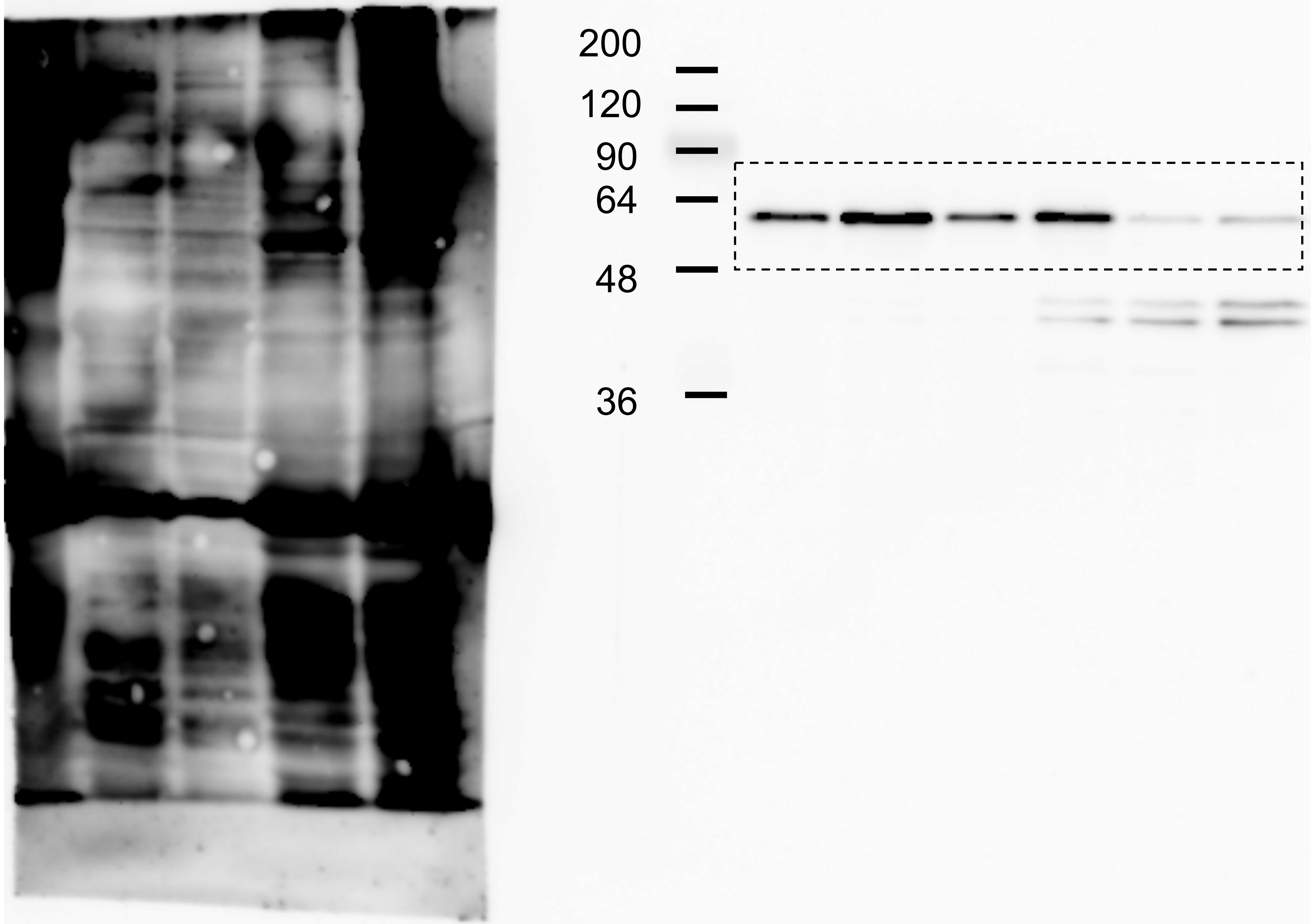

Full unedited gel for Figure 2D (Akt)

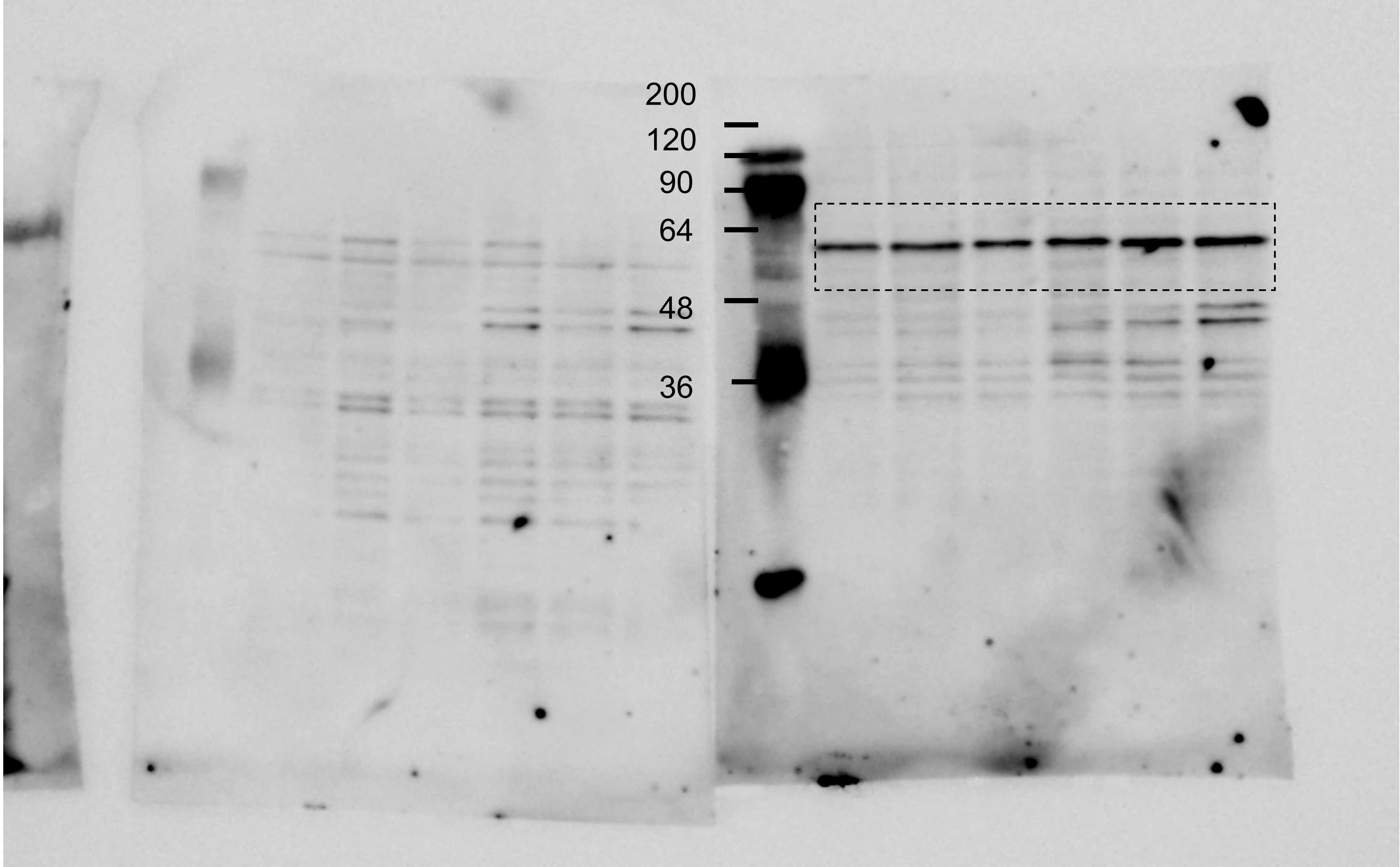

Full unedited gel for Figure 3F (CTH)

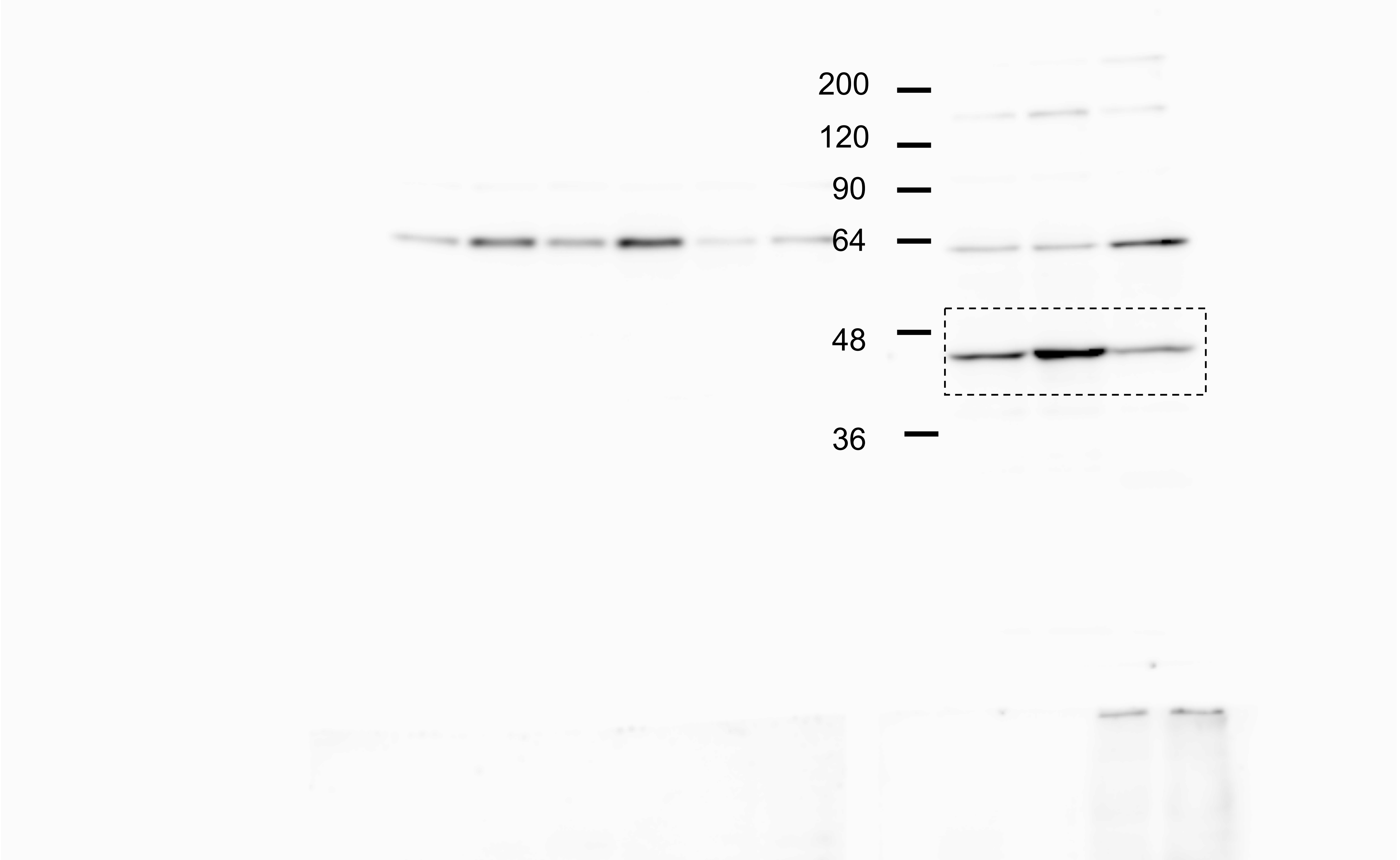

Full unedited gel for Figure 3F (β-actin)

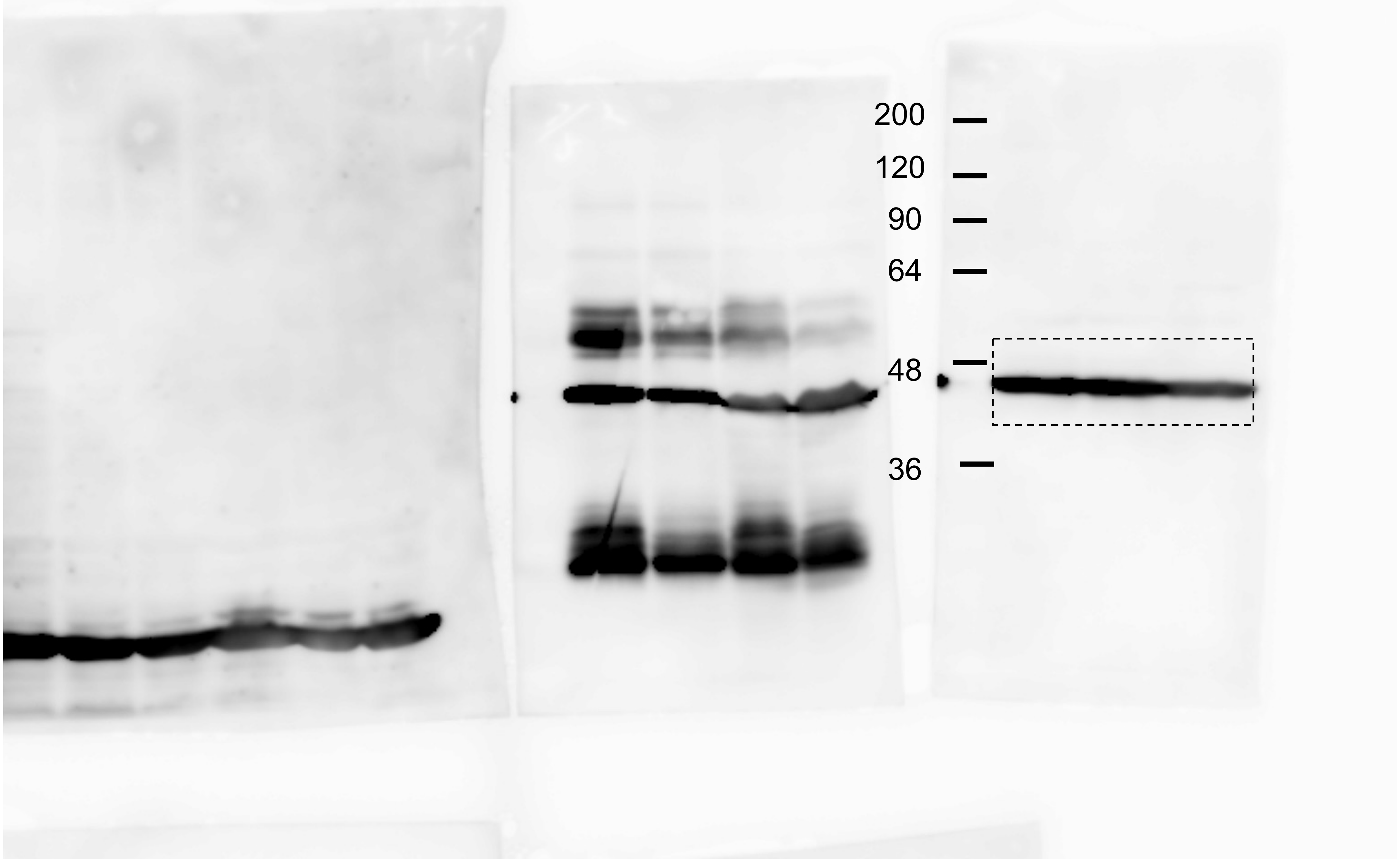

Full unedited gel for Supplemental Figure 4 (Phospho-p65 (S536))

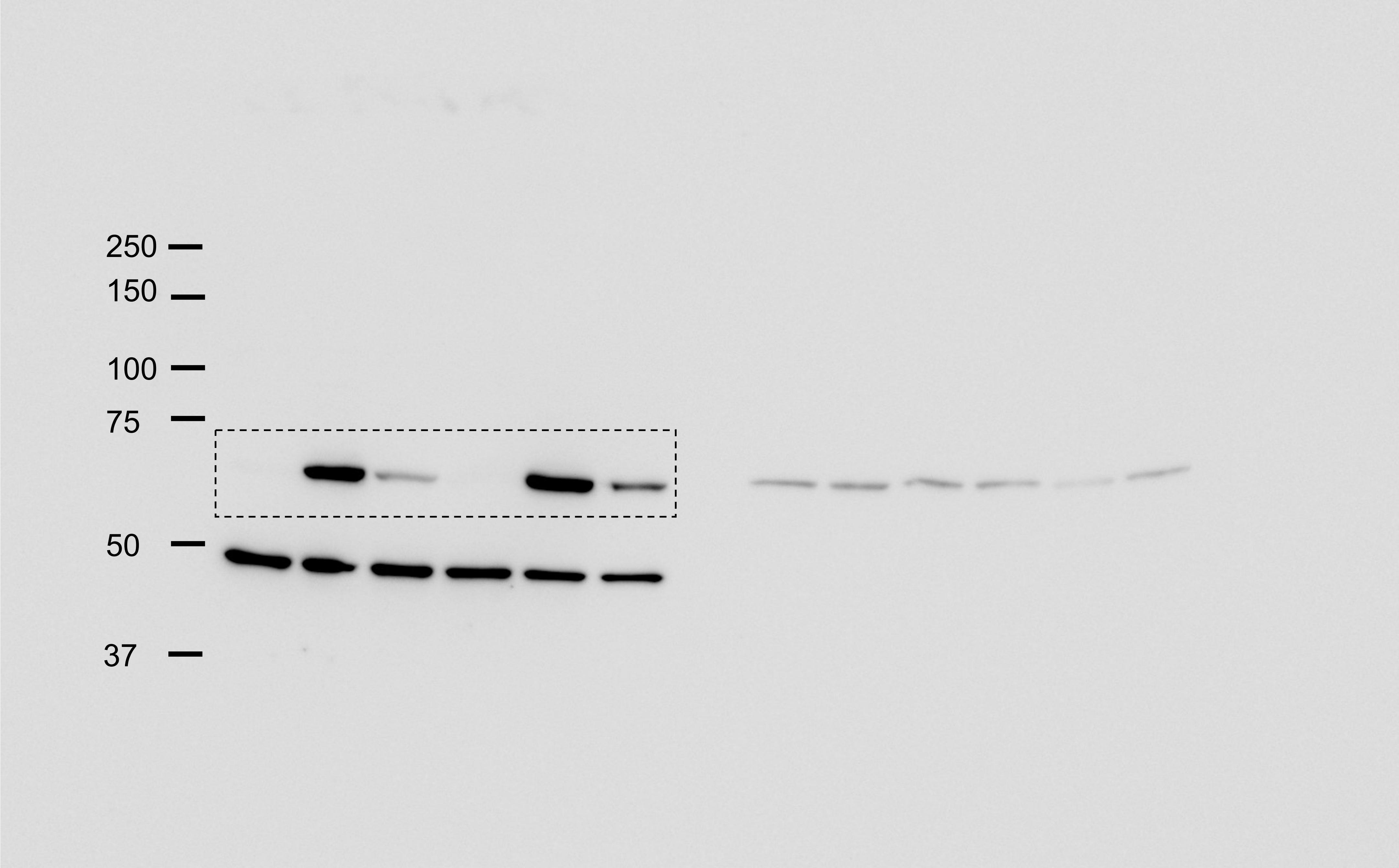

Full unedited gel for Supplemental Figure 4 (p65)

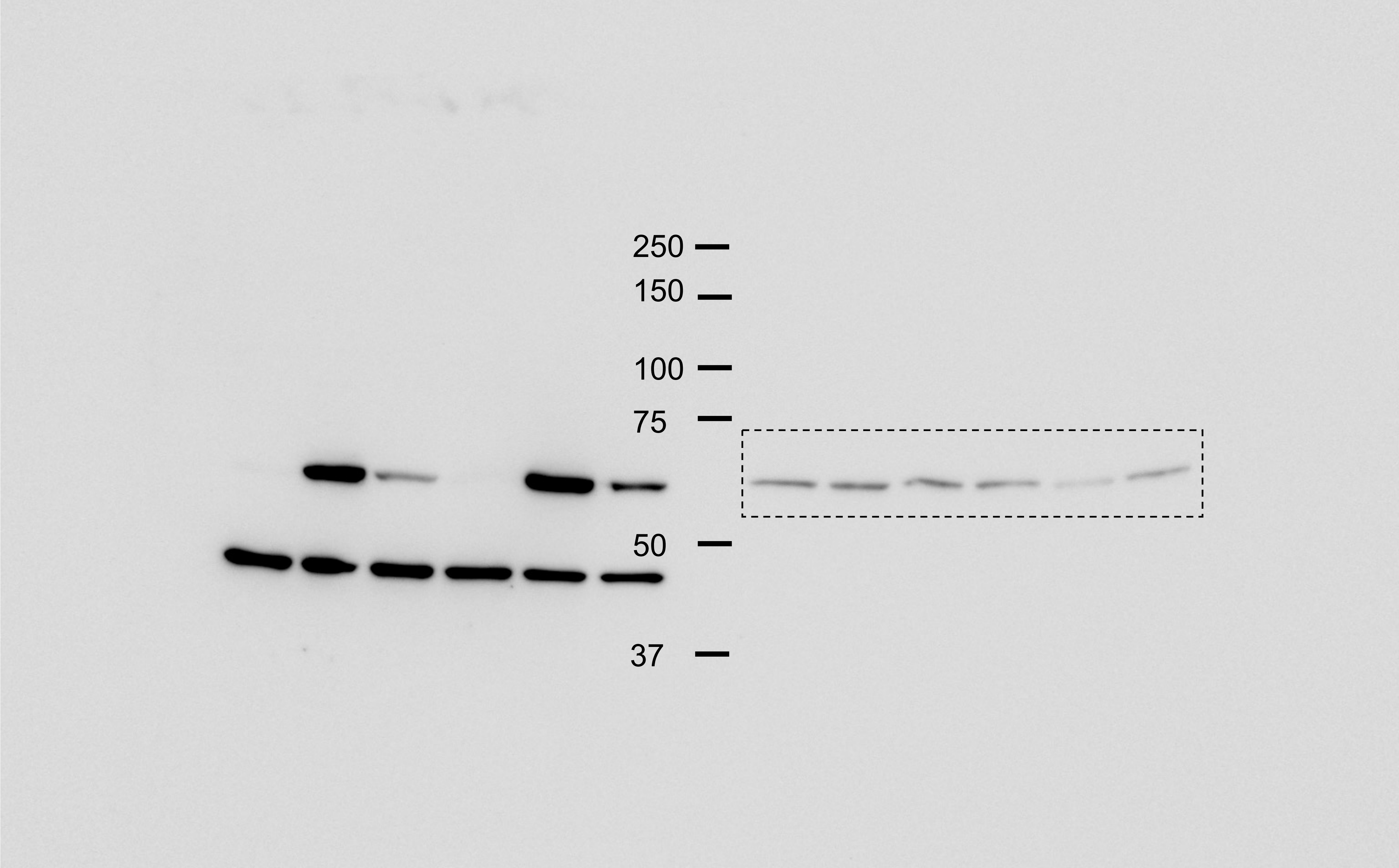

Full unedited gel for Supplemental Figure 4 (p65 (Nuclear fraction))

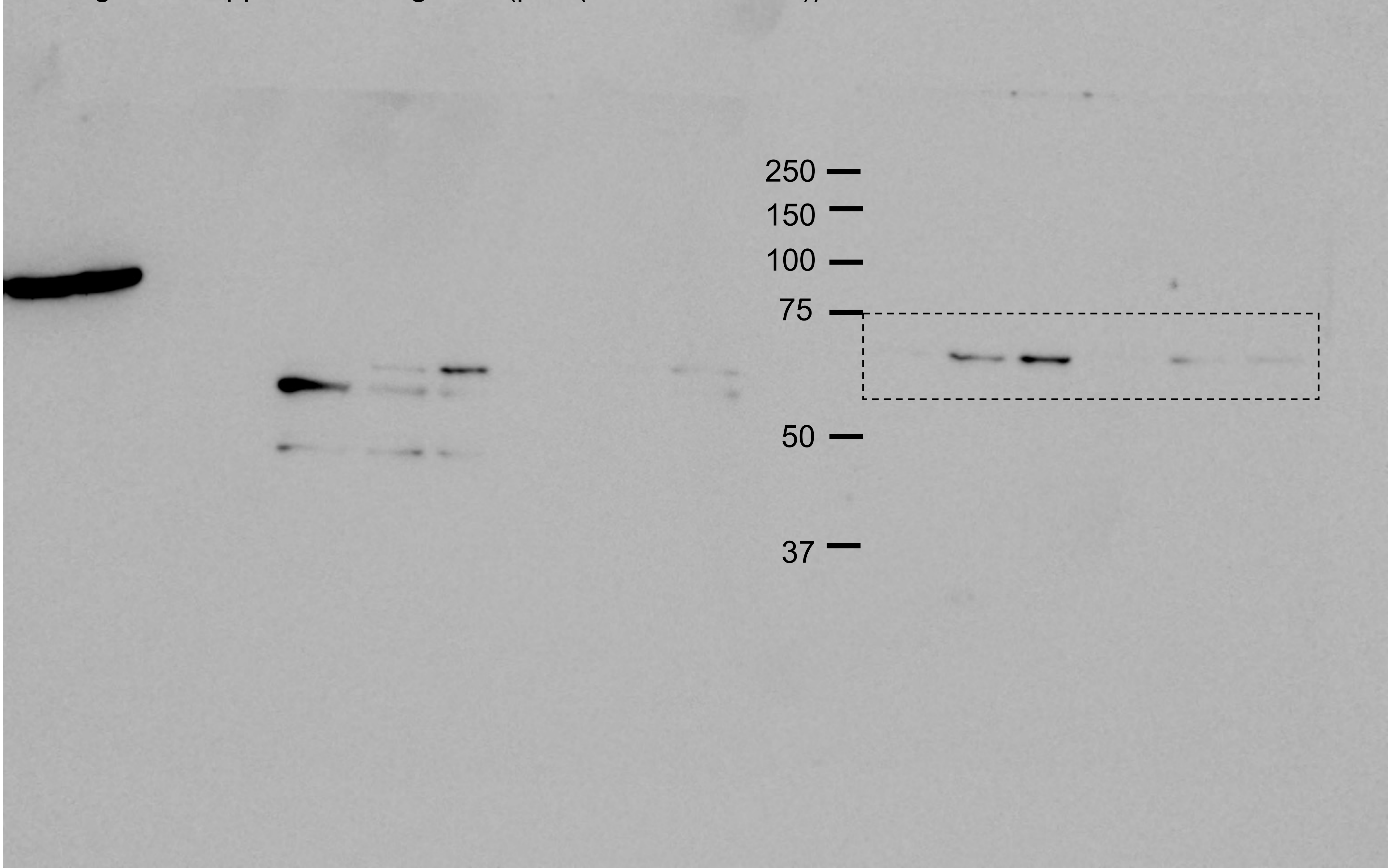

Full unedited gel for Supplemental Figure 4 (Nucleoporin)

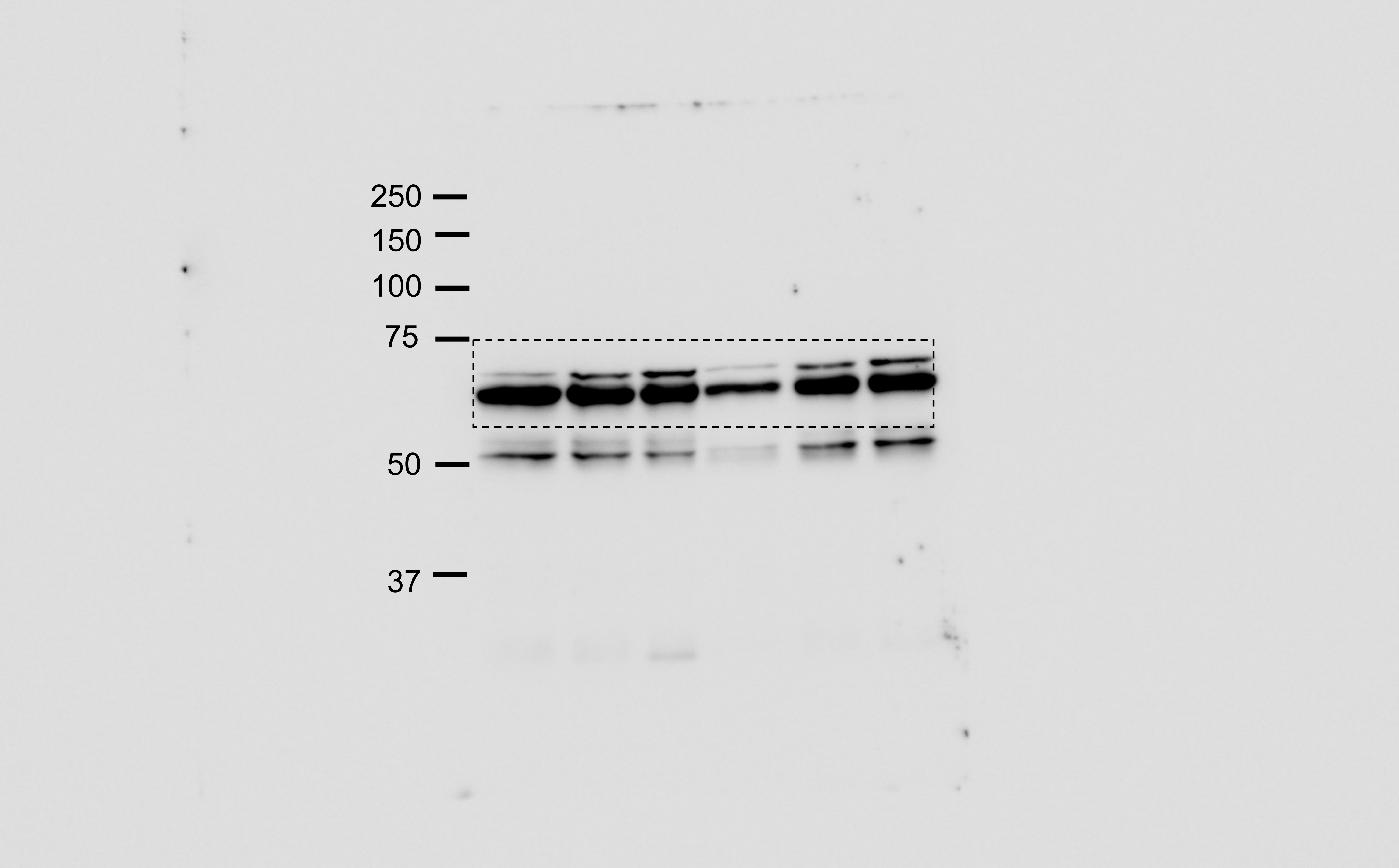

Full unedited gel for Supplemental Figure 16 (Cth)

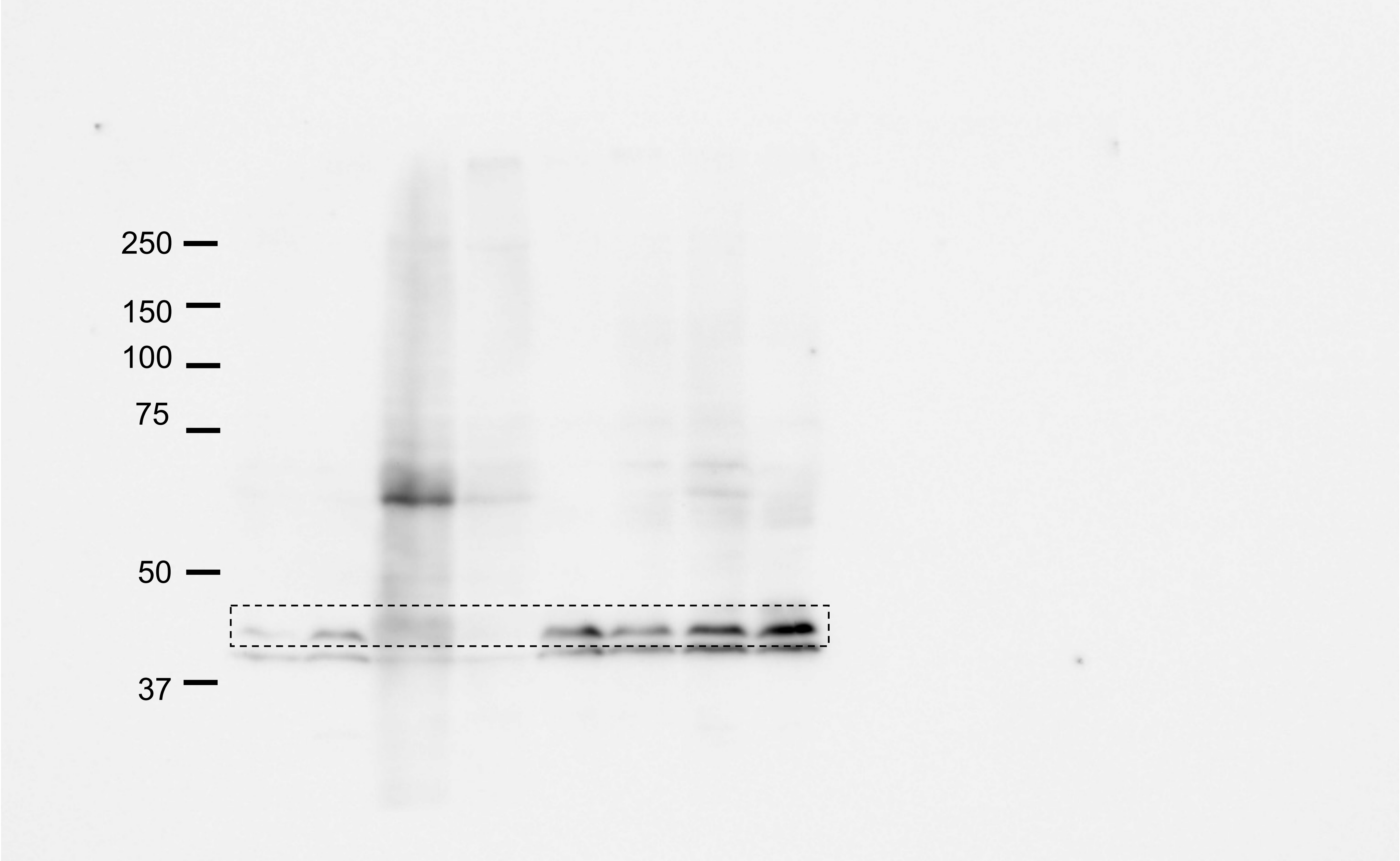

Full unedited gel for Supplemental Figure 16 (β-actin)

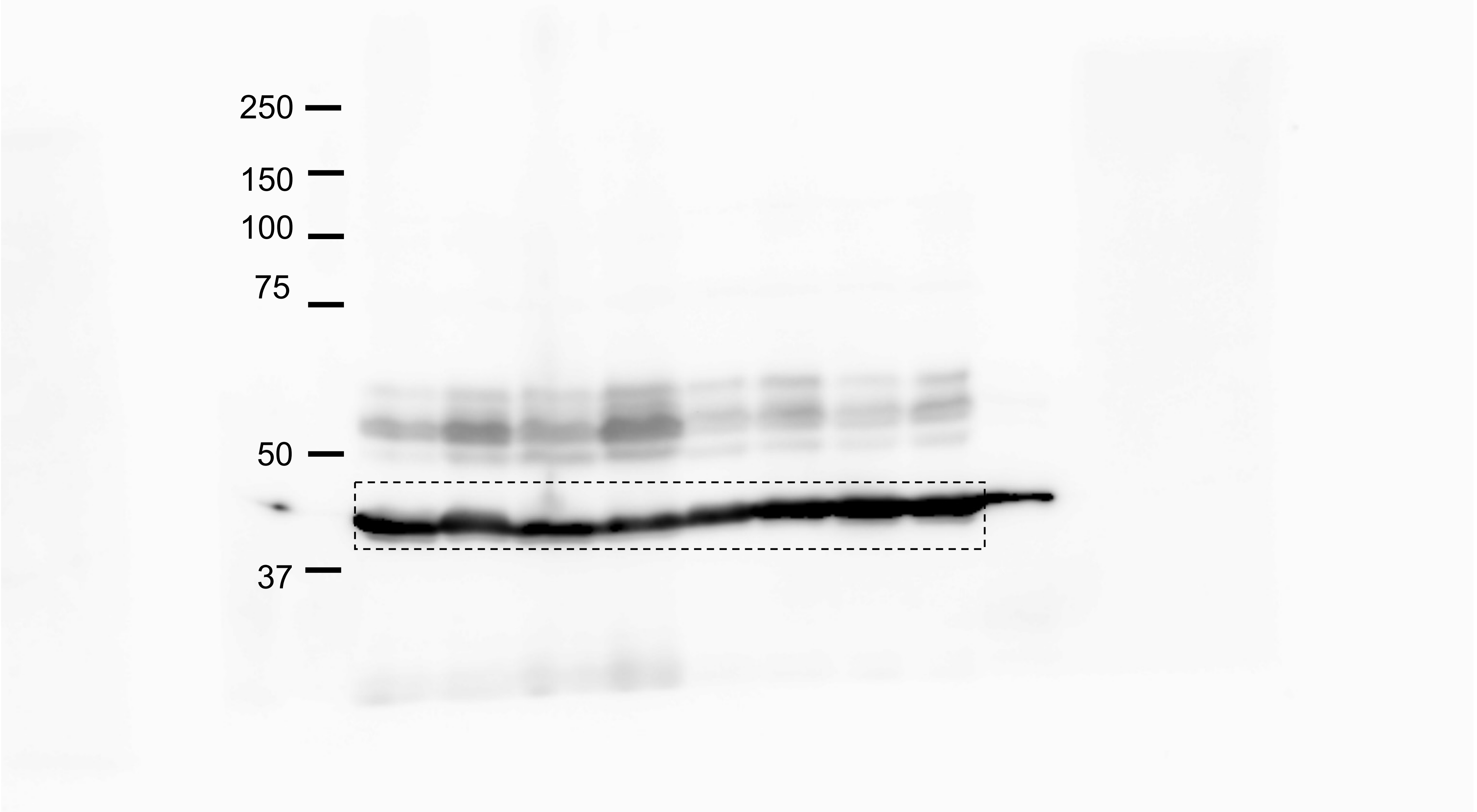

Full unedited gel for Supplemental Figure 18 (Cth)

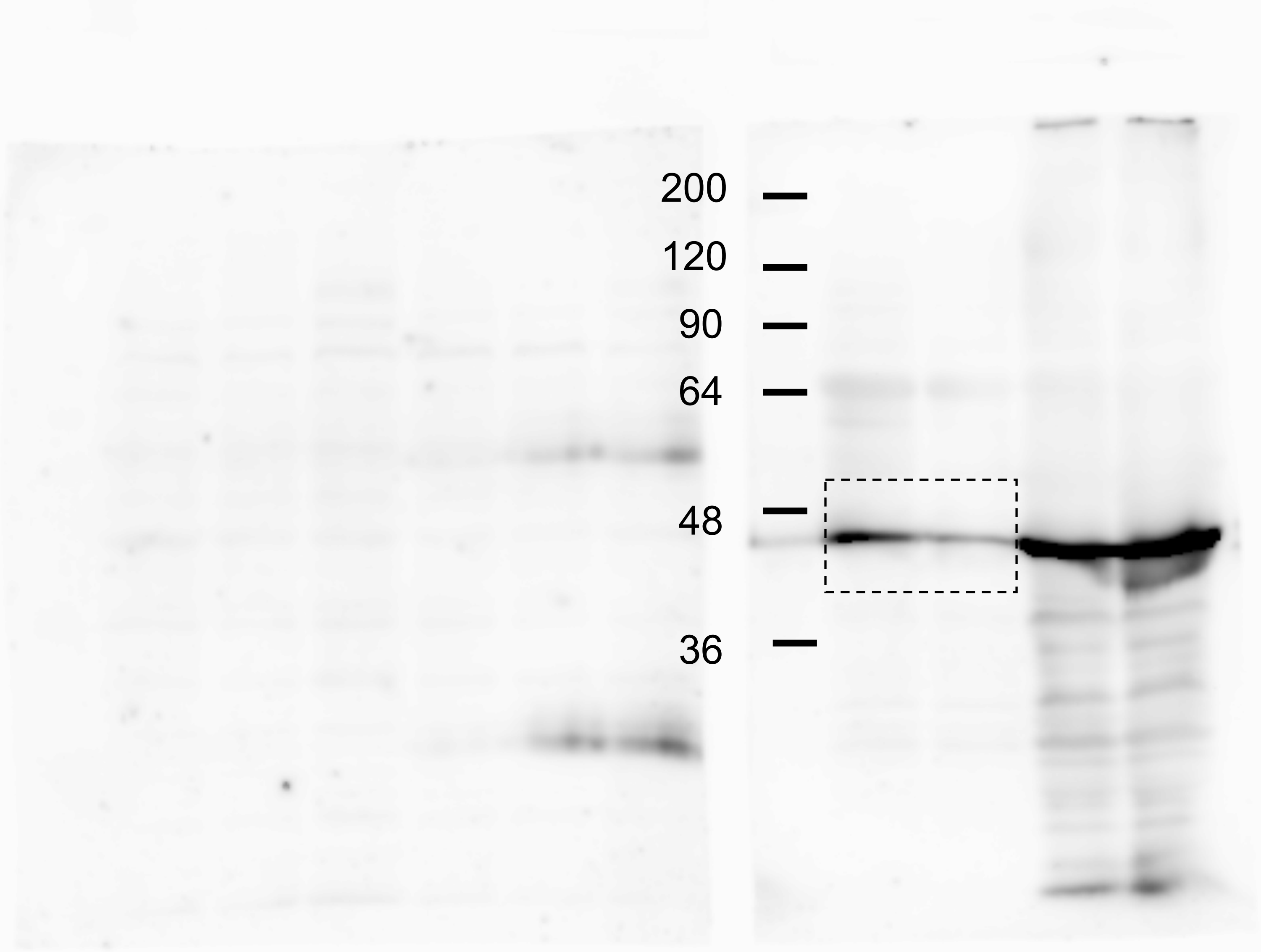

Full unedited gel for Supplemental Figure 18 (β-actin)

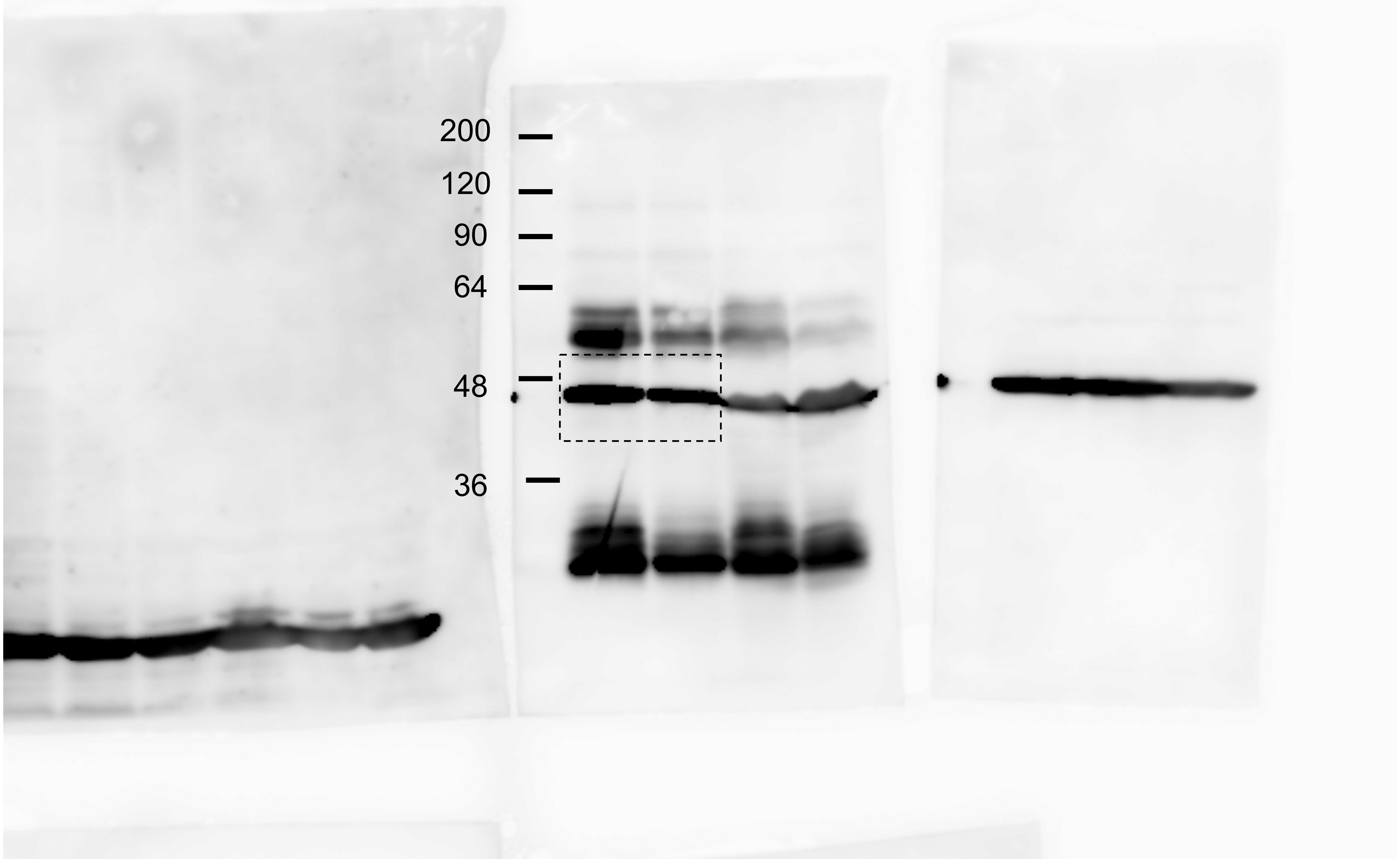

Supplement: Unedited blot and gel images [file jciinsight-9-171371-s131.pdf]
